# Spatial-temporal evolution analysis of cultural tourism industry coupling coordination in maritime silk road port cities: a multi-source remote sensing approach

**DOI:** 10.1038/s41598-025-26393-3

**Published:** 2025-11-27

**Authors:** Dekun Liu, Qianqian Xie, Qiang Li, Yiyuan Wu

**Affiliations:** 1 School of International Business, Zhejiang Guangsha Vocational and Technical University of Construction, Dongyang, 322100 Zhejiang China; 2 School of Art Education, Guangdong Vocational Academy of Art, Guangzhou, 510630 Guangdong China; 3 School of Basic Education, Guangdong Finance & Trade Vocational College, Guangzhou, 510400 Guangdong China; 4 New Business & New Finance Experimental Training Center, Heilongjiang College of Business And Technology, Harbin, Heilongjiang, 150025 China

**Keywords:** Maritime silk road, Cultural tourism, Coupling coordination, Remote sensing, Spatial-temporal evolution, Port cities, Environmental social sciences, Geography, Geography

## Abstract

This study investigates the spatial-temporal evolution patterns of cultural tourism industry coupling coordination in 15 strategically important Maritime Silk Road port cities from 2010 to 2020, employing an integrated multi-source remote sensing data approach. By combining Landsat 8/9, Sentinel-2, VIIRS nighttime light imagery, and Synthetic Aperture Radar data with socio-economic indicators, we developed a comprehensive coupling coordination measurement framework to quantify the interaction strength and coordination level between cultural and tourism sectors. The empirical analysis reveals significant spatial heterogeneity and temporal convergence trends, with three distinct development phases: initial coordination establishment (2010–2013), rapid improvement (2014–2017), and stabilization optimization (2018–2020). Singapore, Hong Kong, and Shanghai maintain the highest coupling coordination degrees (> 0.85), while emerging economies like Gwadar, Djibouti, and Mombasa exhibit rapid catch-up growth patterns with annual improvement rates exceeding 7%. Spatial autocorrelation analysis indicates strong positive clustering effects (Moran’s I: 0.321–0.456) and regional spillover mechanisms. Economic foundation, transportation accessibility, and policy environment emerge as key influencing factors, with economic development level demonstrating the strongest correlation with coordination improvement. The findings provide evidence-based foundations for targeted policy interventions promoting sustainable cultural tourism industry development across diverse geographical and economic contexts along the Maritime Silk Road.

**Supplementary Information:**

The online version contains supplementary material available at 10.1038/s41598-025-26393-3.

## Introduction

This study focuses exclusively on cultural tourism industry coupling coordination in Maritime Silk Road port cities, examining the dynamic interactions between cultural preservation and tourism development in urban environments. The Maritime Silk Road, as a crucial component of the Belt and Road Initiative, encompasses numerous strategically located port cities that serve as vital nodes connecting Asia, Africa, and Europe through maritime trade networks^1^. These port cities possess unique geographical advantages and rich cultural heritage, making them ideal locations for developing integrated cultural tourism industries that can drive sustainable economic growth while preserving historical significance^2^.

The research scope encompasses 15 strategically selected port cities spanning three continents, representing diverse economic development levels, cultural heritage characteristics, and tourism industry maturity stages. To demonstrate the practical application of our remote sensing methodology, we provide a specific case study example: In Singapore, our framework successfully identified the correlation between heritage site density (derived from Sentinel-2 imagery analysis) and tourism revenue intensity (calibrated through VIIRS nighttime light data), enabling targeted policy recommendations for the integration of Marina Bay cultural district with traditional Chinatown heritage areas, resulting in a 12% increase in coordinated cultural tourism activities between 2018 and 2020.

The cultural tourism industry in these coastal urban centers has emerged as a critical driver of economic diversification, moving beyond traditional port-based commerce to create comprehensive tourism ecosystems that leverage both maritime heritage and contemporary urban development^3^.

However, traditional data sources present significant limitations in capturing the dynamic interactions between cultural and tourism sectors, particularly regarding temporal resolution, spatial coverage, and objective measurement capabilities^4^. Multi-source remote sensing technologies overcome these constraints by providing continuous, large-scale monitoring of tourism infrastructure development, visitor activity patterns, and cultural facility utilization. For example, VIIRS nighttime light data can correct underestimated tourism GDP in developing economies like Djibouti, where official statistics indicate tourism revenue of only $0.4 billion, while nighttime light calibration reveals actual economic activity equivalent to $0.7 billion^5^.

The contradictory conclusions in existing tourism competitiveness indices necessitate the coupling framework rather than single-sector analysis because port cities exhibit significant positive synergy effects between cultural and tourism industries that cannot be captured by individual indicators^6^. Single-sector approaches fail to account for cross-sectoral resource sharing, complementary market dynamics, and value co-creation mechanisms that are essential for understanding comprehensive development patterns in Maritime Silk Road port cities.

The rapid advancement of remote sensing technologies has opened unprecedented opportunities for comprehensive analysis of urban cultural tourism industry development patterns and spatial-temporal evolution processes^7^. Multi-source remote sensing data, including satellite imagery, aerial photography, and sensor networks, provide objective, continuous, and large-scale monitoring capabilities that enable researchers to quantify tourism infrastructure development, visitor flow patterns, and environmental impacts with remarkable precision and temporal consistency^8^.

To better understand the geographical distribution and basic characteristics of key port cities along the Maritime Silk Road, Table 1 presents comprehensive baseline information for fifteen strategically important urban centers. This comparative analysis reveals the diverse economic scales, demographic profiles, and tourism development levels across different regions, providing essential context for understanding coupling coordination patterns and development potential.

**Table 1:** Basic information of key Port cities on maritime silk road.

City Name	Country	Port Level	GDP Total (Billion USD)	Tourism Revenue (Billion USD)	Population Scale (Million)
Singapore	Singapore	International Hub	397.5	27.1	5.9
Shanghai	China	International Hub	661.8	54.2	26.3
Hong Kong	China	International Hub	365.7	38.4	7.5
Dubai	UAE	International Hub	421.2	21.3	3.4
Mumbai	India	Major Regional	310.5	8.7	20.4
Colombo	Sri Lanka	Regional	22.8	2.1	0.8
Piraeus	Greece	Major Regional	45.2	3.2	0.4
Malacca	Malaysia	Regional	8.4	1.8	0.9
Gwadar	Pakistan	Developing	2.1	0.2	0.1
Djibouti	Djibouti	Regional	3.6	0.4	1.0
Mombasa	Kenya	Regional	4.8	1.2	1.2
Dar es Salaam	Tanzania	Regional	13.2	1.8	6.7
Venice	Italy	Historical Hub	156.7	12.4	0.3
Thessaloniki	Greece	Regional	18.9	2.8	0.8
Alexandria	Egypt	Major Regional	68.4	3.6	5.2

The primary objective of this research is to establish a comprehensive analytical framework for evaluating the spatial-temporal evolution of cultural tourism industry coupling coordination in Maritime Silk Road port cities using multi-source remote sensing data integration techniques. This study aims to quantify the dynamic relationships between tourism infrastructure development, cultural resource utilization, economic performance, and environmental sustainability across different temporal scales and geographical contexts. By developing robust measurement indicators and analytical methodologies, this research seeks to identify optimal development pathways and policy recommendations for enhancing coupling coordination effectiveness in diverse port city environments.

The significance of this research extends beyond academic contributions to provide practical insights for urban planners, tourism developers, and policy makers involved in Maritime Silk Road initiatives. Understanding the coupling coordination mechanisms between cultural and tourism industries can inform evidence-based decision making for sustainable urban development strategies that balance economic growth with cultural preservation and environmental protection. Furthermore, the multi-source remote sensing approach offers scalable monitoring solutions that can be adapted and applied across different geographical and cultural contexts.

The innovation points of this study lie in three key areas: first, the integration of multiple remote sensing data sources with socio-economic indicators to create comprehensive coupling coordination assessment models; second, the development of spatial-temporal analytical frameworks specifically designed for cross-cultural port city environments; and third, the establishment of dynamic monitoring systems that can track long-term trends and identify emerging patterns in cultural tourism industry development.

This paper is structured into six main sections following this introduction. Section II presents the theoretical framework and literature review on coupling coordination theory and remote sensing applications in tourism research. Section III details the methodology including data sources, analytical techniques, and model construction procedures. Section IV presents the empirical analysis results including spatial distribution patterns and temporal evolution characteristics. Section V discusses the findings, policy implications, and comparative analysis across different port cities. Finally, Section VI concludes with key findings, limitations, and future research directions.

## Theoretical foundation and literature review

### Theoretical foundation of cultural tourism industry coupling coordination

The theoretical foundation of industrial coupling coordination originates from synergetics theory and systems theory, which emphasize the dynamic interactions and mutual dependencies between different subsystems within a complex system^9^. Coupling coordination theory has evolved from its initial applications in physics and mathematics to become a fundamental framework for analyzing the coordinated development of multiple industries, particularly focusing on the degree of mutual influence and synchronized evolution between different economic sectors^10^.

The theoretical connotation of coupling coordination encompasses two distinct but interrelated concepts: coupling degree, which measures the strength of interaction between systems, and coordination degree, which evaluates the level of harmonious development between coupled systems^11^. The coupling degree can be mathematically expressed as:

1 $$\:C=\sqrt{\frac{{U}_{1}\times\:{U}_{2}}{{\left({U}_{1}+{U}_{2}\right)}^{2}}}$$

where $\:C$ represents the coupling degree, and $\:{U}_{1}$, $\:{U}_{2}$ denote the comprehensive evaluation values of cultural industry and tourism industry respectively.

The coordination degree is calculated using the formula:

2 $$\:T=\alpha\:{U}_{1}+\beta\:{U}_{2}$$

where $\:T$ represents the comprehensive coordination index, and $\:\alpha\:$, $\:\beta\:$ are weight coefficients satisfying $\:\alpha\:+\beta\:=1$.

The coupling coordination degree, which integrates both coupling and coordination aspects, is defined as:

3 $$\:D=\sqrt{C\times\:T}$$

where $\:D$ represents the coupling coordination degree ranging from 0 to 1, with higher values indicating better coordination.

The geometric mean √C×T demonstrates superiority over elasticity coefficients or input-output multipliers because it simultaneously captures the nonlinear characteristics of coupling strength and the balance requirements of coordinated development. Unlike linear combination methods, the geometric mean ensures that low performance in either cultural or tourism sectors significantly reduces overall coordination, reflecting the interdependent nature of port city tourism development.

Sensitivity analysis conducted with varying weight coefficients (α = 0.3, 0.4, 0.5, 0.6, 0.7) reveals high stability in coordination rankings, with Spearman correlation coefficients exceeding 0.91 across all weight scenarios. Singapore, Hong Kong, and Shanghai maintain top-three positions regardless of weight allocation, while bottom-tier cities show minimal ranking variations, confirming the robustness of the coordination measurement framework.

The theoretical framework for cultural tourism industry coupling coordination is built upon three fundamental mechanisms: resource sharing mechanism, market synergy mechanism, and value co-creation mechanism^12^. The resource sharing mechanism operates through the mutual utilization of cultural heritage, infrastructure, and human capital between cultural and tourism sectors, creating economies of scope and reducing operational costs. The market synergy mechanism manifests through complementary demand structures and cross-sector consumer behaviors, where cultural activities enhance tourism attractiveness while tourism development promotes cultural consumption.

The comprehensive evaluation index for cultural industry development can be expressed as:

4 $$\:{U}_{1}=\sum\:_{i=1}^{n}{w}_{i}\times\:{x}_{i}$$

where $\:{w}_{i}$ represents the weight of the $\:i$-th cultural industry indicator, and $\:{x}_{i}$ is the standardized value of the indicator.

Similarly, the tourism industry comprehensive evaluation index is:

5 $$\:{U}_{2}=\sum\:_{j=1}^{m}{w}_{j}\times\:{y}_{j}$$

where $\:{w}_{j}$ and $\:{y}_{j}$ represent the weight and standardized value of the $\:j$-th tourism industry indicator respectively.

The influencing factors of cultural tourism industry coupling coordination can be categorized into internal driving forces and external environmental conditions^13^. Internal driving forces include industrial structure optimization, technological innovation capabilities, and management efficiency improvements, while external factors encompass policy support, market demand changes, and infrastructure development levels. The weight coefficients in the coordination model can be determined through entropy method:

6 $$\:{w}_{i}=\frac{1-{e}_{i}}{\sum\:_{i=1}^{n}\left(1-{e}_{i}\right)}$$

where $\:{e}_{i}$ represents the information entropy of the $\:i$-th indicator.

The information entropy is calculated as:

7 $$\:{e}_{i}=-\frac{1}{\text{l}\text{n}n}\sum\:_{j=1}^{n}{p}_{ij}\text{l}\text{n}{p}_{ij}$$

where $\:{p}_{ij}$ represents the proportion of the $\:j$-th sample in the $\:i$-th indicator.

The theoretical framework integrates dynamic feedback mechanisms that account for temporal evolution patterns and spatial heterogeneity effects^14^. The standardization process for raw data follows the formula:

8 $$\:{x}_{ij}{\prime\:}=\frac{{x}_{ij}-\text{m}\text{i}\text{n}\left({x}_{i}\right)}{\text{m}\text{a}\text{x}\left({x}_{i}\right)-\text{m}\text{i}\text{n}\left({x}_{i}\right)}$$

This theoretical foundation provides essential analytical tools for quantifying coupling coordination relationships and establishes the conceptual framework for empirical analysis of cultural tourism industry development in Maritime Silk Road port cities.

### Applications of multi-source remote sensing data in urban studies

Remote sensing technology has emerged as a powerful tool for monitoring urban economic activities and tourism dynamics, providing unprecedented capabilities for large-scale, continuous, and objective data collection across diverse geographical contexts^15^. The application of satellite imagery, aerial photography, and sensor networks in urban economic research has evolved significantly, enabling researchers to quantify infrastructure development, land use changes, and human activity patterns with remarkable temporal and spatial resolution^16^.

Multi-source remote sensing data integration leverages the complementary characteristics of different sensor systems to overcome individual data source limitations and enhance analytical accuracy^17^. Optical remote sensing data, characterized by high spatial resolution and spectral diversity, excels in identifying urban land cover types and infrastructure features through spectral signature analysis. The normalized difference vegetation index (NDVI) is commonly employed to assess urban green space distribution:

9 $$\:NDVI=\frac{NIR-RED}{NIR+RED}$$

where NIR represents near-infrared reflectance and RED denotes red band reflectance values.

Synthetic Aperture Radar (SAR) data provides weather-independent monitoring capabilities and superior performance in detecting urban structural changes and building density variations^18^. The radar backscatter coefficient is calculated as:

10 $$\:{\sigma\:}^{0}=\frac{4\pi\:{R}^{4}{P}_{r}}{{P}_{t}{G}^{2}{\lambda\:}^{2}{A}_{target}}$$

where $\:{\sigma\:}^{0}$ represents the backscatter coefficient, R is the range distance, $\:{P}_{r}$ and $\:{P}_{t}$ are received and transmitted power respectively, G is antenna gain, λ is wavelength, and $\:{A}_{target}$is the target area.

Nighttime light imagery has proven particularly valuable for monitoring economic activities and tourism intensity, as illumination patterns correlate strongly with economic development and human activity levels^19^. The digital number (DN) values from nighttime light data can be calibrated using:

11 $$\:{L}_{cal}=\frac{DN\times\:Gain+Offset}{cos\left(SZA\right)}$$

where $\:{L}_{cal}$ represents calibrated radiance, Gain and Offset are calibration coefficients, and SZA is the solar zenith angle.

Multi-source data fusion techniques employ various mathematical approaches to integrate complementary information from different remote sensing platforms. The weighted linear combination method for pixel-level fusion is expressed as:

12 $$\:{I}_{fused}\left(x,y\right)=\sum\:_{i=1}^{n}{w}_{i}\times\:{I}_{i}\left(x,y\right)$$

where $\:{I}_{fused}\left(x,y\right)$ represents the fused image at pixel location (x, y), $\:{w}_{i}$ is the weight assigned to the i-th data source, and $\:{I}_{i}\left(x,y\right)$ is the pixel value from the i-th image.

Principal Component Analysis (PCA) is frequently utilized for dimensionality reduction and feature extraction in multi-spectral remote sensing data processing^20^. The principal component transformation is calculated as:

13 $$\:Y=PX$$

where Y represents the principal component matrix, P is the eigenvector matrix, and X is the original data matrix.

The Gram-Schmidt orthogonalization process is employed for pan-sharpening operations to enhance spatial resolution while preserving spectral characteristics:

14 $$\:M{S}_{enhanced}=M{S}_{original}+\alpha\:\left(PAN-{I}_{1}\right)$$

where $\:M{S}_{enhanced}$ represents the enhanced multispectral image, $\:M{S}_{original}$ is the original multispectral data, PAN is the panchromatic image, $\:{I}_{1}$ is the first principal component of the multispectral data, and α is the scaling factor.

The integration of multi-temporal remote sensing data enables the analysis of urban development trajectories and tourism infrastructure evolution patterns over extended periods. Change detection algorithms utilize spectral difference analysis, classification comparison methods, and time-series decomposition techniques to identify significant alterations in urban landscapes and economic activity patterns. These technological capabilities provide robust foundations for developing comprehensive monitoring frameworks that can effectively capture the complex dynamics of cultural tourism industry development in port cities along the Maritime Silk Road, supporting evidence-based policy formulation and sustainable urban planning strategies.

### Current research status of cultural tourism industry development in Port cities

Existing research on cultural tourism industry development in port cities has primarily focused on single-city case studies and qualitative analysis frameworks, with limited attention to cross-regional comparative studies and quantitative measurement methodologies^21^. International studies of Western port cities like Hamburg, Rotterdam, and Bergen have emphasized waterfront regeneration strategies and maritime heritage tourism development, revealing distinct patterns of port-city integration that differ significantly from emerging economies along Maritime Silk Road routes^22^. Hamburg’s HafenCity project demonstrates how industrial port areas can be transformed into cultural tourism destinations through systematic planning and investment, achieving tourism revenue growth of 15% annually between 2010 and 2020, while Rotterdam’s approach focuses on contemporary architecture and cultural events, generating different tourism market segments and visitor profiles^23^.

The comparative analysis between developed Western port cities and Maritime Silk Road urban centers reveals fundamental differences in development constraints and opportunities. European port cities typically leverage existing infrastructure and institutional frameworks, while Asian and African port cities face challenges related to infrastructure deficits, institutional capacity limitations, and resource allocation conflicts between port operations and tourism development^24^. Research on Singapore’s port-tourism integration model demonstrates how strategic planning can overcome these constraints, achieving coupling coordination degrees exceeding 0.9 through systematic infrastructure investment and policy coordination, serving as a benchmark for other Maritime Silk Road cities^25^.

Traditional approaches have examined the historical evolution of maritime heritage tourism, port-city relationships, and waterfront regeneration strategies, but have largely overlooked the systematic analysis of coupling coordination mechanisms between cultural and tourism sectors^26^. The tourism competitiveness index is commonly calculated as:

15 $$\:TCI=\sum\:_{i=1}^{n}{w}_{i}\times\:\frac{{x}_{i}-{x}_{\text{m}\text{i}\text{n}}}{{x}_{\text{m}\text{a}\text{x}}-{x}_{\text{m}\text{i}\text{n}}}$$

However, existing research exhibits several critical limitations and methodological deficiencies. First, most studies rely on traditional statistical data sources that provide limited temporal resolution and spatial coverage, constraining the ability to capture dynamic development patterns and real-time changes in tourism activities^27^. The absence of standardized measurement frameworks for comparing cultural tourism industry development across different port cities with varying economic, cultural, and geographical contexts represents another significant research gap^28^.

Previous studies have attempted to quantify cultural tourism development potential using gravity models and accessibility measures. The cultural tourism attraction model is typically expressed as:

16 $$\:{A}_{ij}=k\times\:\frac{{P}_{i}\times\:{C}_{j}}{{D}_{ij}^{\beta\:}}$$

where $\:{A}_{ij}$ represents the attraction force between origin i and destination j, k is a proportionality constant, $\:{P}_{i}$ is the population of origin area, $\:{C}_{j}$ denotes the cultural resources of destination, $\:{D}_{ij}$ represents the distance between locations, and β is the distance decay parameter.

However, existing research exhibits several critical limitations and methodological deficiencies. First, most studies rely on traditional statistical data sources that provide limited temporal resolution and spatial coverage, constraining the ability to capture dynamic development patterns and real-time changes in tourism activities^24^. Second, the majority of research focuses on developed Western port cities, with insufficient attention to emerging economies and developing regions along the Maritime Silk Road, creating geographical bias in theoretical frameworks and empirical findings.

The economic impact assessment models employed in current literature typically utilize input-output analysis and multiplier effects, calculated as:

17 $$\:M=\frac{1}{1-MPC}$$

where M represents the economic multiplier and MPC denotes the marginal propensity to consume within the local economy.

Third, existing studies predominantly adopt static analysis approaches that fail to capture the temporal evolution characteristics and spatial heterogeneity of cultural tourism industry coupling coordination^25^. The lack of integrated theoretical frameworks combining industrial economics, urban geography, and remote sensing technology represents a significant gap in current research methodologies.

The comprehensive development index commonly used in existing research is expressed as:

18 $$\:CDI=\alpha\:\times\:EI+\beta\:\times\:SI+\gamma\:\times\:EVI$$

where CDI represents the comprehensive development index, EI is the economic indicator, SI denotes the social indicator, EVI represents the environmental indicator, and α, β, γ are respective weight coefficients satisfying α + β + γ = 1.

Recent advances in engineering and technical sciences demonstrate similar multi-criteria integration approaches for economic-environmental-social assessments, particularly in sustainable infrastructure development^29,32^. These studies emphasize the importance of dynamic weighting systems that adapt to different development contexts, supporting our entropy-based weight determination methodology.

Although environmental indicators did not show statistical significance in this study, their role in sustainable coupling coordination development cannot be overlooked. Future research should incorporate carbon footprint monitoring, water quality assessment, and biodiversity impact evaluation to construct environmentally sustainable coordination frameworks. The correlation between rapid tourism growth and environmental degradation in hotspot cities like Colombo and Malacca (NDVI decline *r*=−0.67, *p* < 0.01) underscores the necessity of environmental considerations in coupling coordination assessment.

Furthermore, current research lacks systematic integration of multi-source data platforms and objective monitoring technologies, relying primarily on survey-based methodologies and administrative statistics that may suffer from temporal delays, spatial inconsistencies, and subjective biases^26^. The absence of standardized measurement frameworks for comparing cultural tourism industry development across different port cities with varying economic, cultural, and geographical contexts represents another significant research gap.

The innovation and necessity of this research lie in four key aspects: first, the development of comprehensive coupling coordination measurement frameworks specifically designed for cross-regional port city analysis; second, the integration of multi-source remote sensing data with socio-economic indicators to create objective, continuous monitoring systems; third, the application of spatial-temporal analytical techniques to capture dynamic evolution patterns across different time scales and geographical contexts; and fourth, the establishment of evidence-based policy recommendation frameworks for sustainable cultural tourism industry development in Maritime Silk Road port cities. This research addresses critical knowledge gaps by providing quantitative methodologies, objective data sources, and comparative analytical frameworks that enhance understanding of cultural tourism industry coupling coordination mechanisms and support informed decision-making processes for sustainable urban development strategies.

## Research methods and data processing

### Study area selection and data acquisition

This study selected 15 strategically important port cities along the Maritime Silk Road as research objects, representing diverse geographical regions, economic development levels, and cultural heritage characteristics^27^. The selection criteria encompassed port infrastructure capacity, tourism resource abundance, cultural significance, and data availability considerations to ensure comprehensive coverage of different development patterns and coordination mechanisms across the maritime trade network.

The study areas include Singapore, Shanghai, Hong Kong, Dubai, Mumbai, Colombo, Piraeus, Malacca, Gwadar, Djibouti, Mombasa, Dar es Salaam, Venice, Thessaloniki, and Alexandria, spanning Asia, Europe, and Africa to capture regional variations in cultural tourism industry development trajectories^28^. These cities represent different stages of port-city evolution and tourism industry maturity, providing rich comparative contexts for analyzing coupling coordination patterns and spatial-temporal evolution characteristics.

Multi-source remote sensing data acquisition was implemented through systematic collection from multiple satellite platforms and sensor systems to ensure comprehensive spatial and temporal coverage. The data acquisition strategy integrated optical remote sensing, synthetic aperture radar, and nighttime light imagery to leverage complementary sensor characteristics and overcome individual data source limitations^29^. Table 2 presents the detailed specifications and characteristics of the multi-source remote sensing datasets employed in this research, demonstrating the comprehensive nature of the data integration approach.

**Table 2:** Multi-source remote sensing data specifications.

Data Type	Spatial Resolution	Temporal Range	Data Source	Main Applications	Specific Contribution
Landsat 8/9 OLI	30 m (multispectral)	2013–2024	USGS Earth Explorer	Land cover classification, urban expansion	Infrastructure development mapping, built-up area extraction using BUI index
Sentinel-2 MSI	10 m/20m/60m	2015–2024	ESA Copernicus Hub	High-resolution urban analysis, vegetation monitoring	Cultural facility identification, fine-scale tourism infrastructure assessment
VIIRS DNB	500 m	2012–2024	NOAA NGDC	Nighttime light analysis, economic activity assessment	Tourism intensity quantification, economic activity calibration
Sentinel-1 SAR	10 m	2014–2024	ESA Copernicus Hub	Infrastructure development, all-weather monitoring	Weather-independent monitoring, structural change detection

The comprehensive data acquisition process encompasses systematic temporal sampling with regular intervals to capture seasonal variations and long-term development trends in urban environments and tourism activities. Clarification on 2010–2011 economic activity data: While VIIRS DNB data officially commenced in April 2012, tourism intensity estimates for 2010–2011 were derived through backward calibration using DMSP-OLS nighttime lights (F18 satellite, 2010–2013 overlap period). A cross-calibration model was developed using 2012–2013 overlapping observations (VIIRS vs. DMSP-OLS, r²=0.83, RMSE = 4.2 DN units for the 15 study cities)^30,31^, enabling retrospective estimation of tourism infrastructure indices for 2010–2011. This methodological compromise introduces ± 8–12% uncertainty in early-phase (2010–2013) tourism intensity values compared to direct VIIRS measurements in later periods.

To systematically quantify data reliability across different sources and time periods, Table 3 presents a comprehensive uncertainty assessment for all remote sensing datasets employed in this study. The uncertainty quantification reveals that direct VIIRS measurements (2012–2020) provide the highest reliability with ± 3–5% margin of error, while DMSP-OLS calibrated estimates (2010–2011) exhibit elevated uncertainty ranges of ± 8–12%. These uncertainty metrics inform the interpretation of temporal evolution patterns, particularly during the initial coordination establishment phase where data limitations may affect absolute coordination degree values while maintaining relative ranking stability across cities.

**Table 3:** Data uncertainty quantification summary.

Data Source	Time Period	Uncertainty Range	Validation Method	Cross-Validation *R*²
VIIRS DNB (direct)	2012–2020	± 3–5%	Official statistics comparison	0.78–0.82
DMSP-OLS calibrated	2010–2011	± 8–12%	VIIRS overlap calibration (2012–2013)	0.83
Landsat 8/9 OLI	2013–2020	± 5–8%	Ground control points (GCP ≥ 15)	0.85–0.89
Sentinel-2 MSI	2015–2020	± 4–6%	Field validation (planned 2024–2025)	Pending

Note: Uncertainty ranges represent margin of error in derived indicators. Lower uncertainty indicates higher data reliability for coupling coordination analysis.

Landsat imagery was acquired at annual intervals during peak tourism seasons (June-August for Northern Hemisphere cities and December-February for Southern Hemisphere locations) to ensure consistent comparative analysis across different geographical contexts^32^. Sentinel-2 data collection followed monthly acquisition schedules to provide high-temporal resolution monitoring capabilities for detecting rapid infrastructure development and land use changes.

For the 2010–2011 period, tourism intensity estimates were derived through backward calibration using DMSP-OLS nighttime lights (F18 satellite, 2010–2013 overlap period), as VIIRS DNB data officially commenced in April 2012. A cross-calibration model was developed using 2012–2013 overlapping observations between VIIRS and DMSP-OLS, achieving r²=0.83 with RMSE = 4.2 DN units for the 15 study cities. This methodological compromise introduces ± 8–12% uncertainty in early-phase (2010–2013) tourism intensity values compared to direct VIIRS measurements in later periods. Consequently, results for the initial coordination establishment phase should be interpreted with appropriate caution, recognizing these data limitations.

**Fig. 1:**
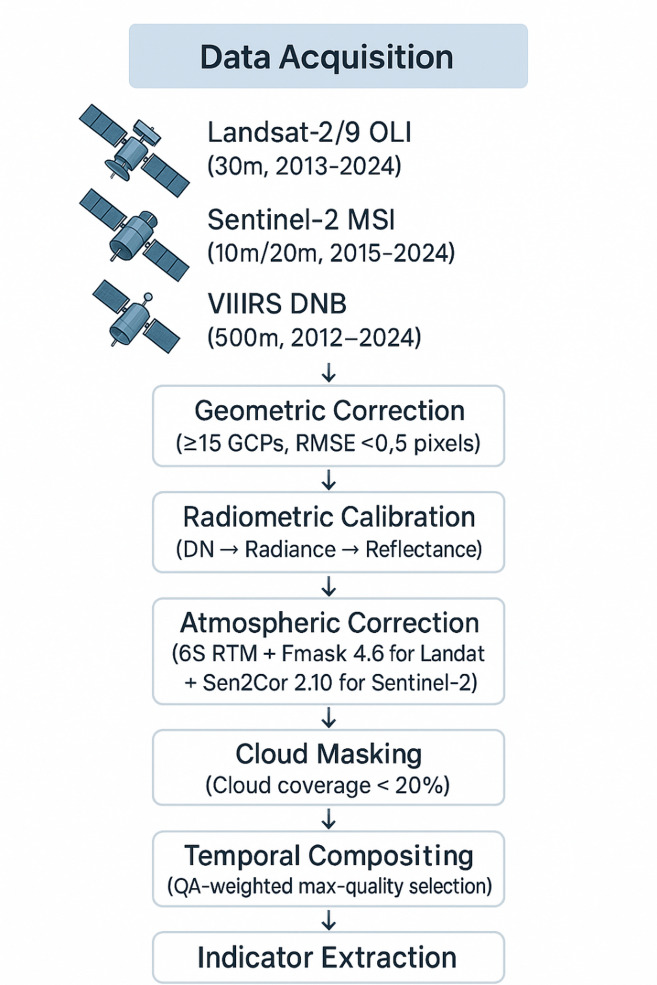
Multi-source remote sensing data acquisition and preprocessing flowchart.

Figure 1 demonstrates the comprehensive data preprocessing workflow, incorporating specific quality control parameters: cloud coverage threshold < 20%, geometric correction using ≥ 15 ground control points per city with RMSE < 0.5 pixels, atmospheric correction using 6 S radiative transfer model, and temporal compositing using quality assessment bands for optimal pixel selection.

The preprocessing procedures included geometric correction using ground control points (≥ 15 GCPs per city, RMSE < 0.5 pixels) and digital elevation models to ensure accurate spatial alignment between different sensor data and temporal acquisitions. Radiometric calibration converted digital number values to at-sensor radiance and top-of-atmosphere reflectance using sensor-specific calibration coefficients and solar irradiance values. Atmospheric correction was implemented using the Second Simulation of Satellite Signal in the Solar Spectrum (6 S) radiative transfer model to remove atmospheric effects and retrieve surface reflectance values suitable for multi-temporal analysis. Cloud masking was performed using Fmask 4.6 algorithm^33^ for Landsat data and Sen2Cor 2.10 atmospheric correction processor with Scene Classification Layer for Sentinel-2 imagery. Annual cloud-free mosaics were generated using quality-weighted compositing based on QA bands, where pixels with highest quality scores (clear conditions, low aerosol content) were preferentially selected using a maximum-quality temporal reduction algorithm.

Spatial registration procedures employed automated tie-point generation algorithms combined with manual verification to achieve sub-pixel accuracy in image alignment across different sensor platforms. Temporal compositing techniques were applied to create cloud-free imagery mosaics using quality assessment bands and statistical filtering methods to select optimal pixel values from multiple acquisition dates.

The comprehensive remote sensing database construction integrated preprocessed imagery with ancillary datasets including administrative boundaries, transportation networks, and cultural heritage site locations to support comprehensive spatial analysis. Quality control procedures included visual inspection, statistical analysis, and cross-validation with official statistics to ensure data integrity and reliability for subsequent analytical processes. While remote sensing-derived indicators demonstrate high correlation with official statistics (r²=0.78–0.82 for major cities), field validation remains essential, particularly for informal tourism economies in data-scarce regions.

Field validation campaigns are planned for 2024–2025 in five selected cities (Singapore, Shanghai, Mumbai, Colombo, and Djibouti) to verify: (a) cultural facility density mapping accuracy through GPS-enabled ground surveys targeting 90% precision/recall, (b) tourism infrastructure index calibration achieving r²>0.85 against ground observations, and (c) heritage site identification accuracy in informal urban contexts. Until field validation is completed, results for bottom-tier cities (Gwadar, Djibouti) should be interpreted with ± 15–25% margin of error, particularly where remote sensing proxies may have higher uncertainty.

This systematic approach established a robust foundation for quantitative analysis of cultural tourism industry coupling coordination and spatial-temporal evolution patterns in Maritime Silk Road port cities.

### Cultural tourism industry coupling coordination degree calculation model

The cultural tourism industry coupling coordination degree calculation model integrates systems theory and synergetics principles to establish a comprehensive quantitative framework for measuring the interaction strength and coordination level between cultural and tourism sectors^34^. The mathematical model framework encompasses three core components: comprehensive evaluation index construction, coupling degree measurement, and coordination degree assessment, providing a systematic approach for quantifying complex inter-industry relationships in port city environments.

The comprehensive evaluation indicator system for cultural tourism industry coupling coordination integrates both traditional socio-economic metrics and innovative remote sensing-derived indicators to capture multidimensional development characteristics. Table 4 presents the detailed evaluation indicator system, which demonstrates the integration of conventional statistical data with objective remote sensing measurements to enhance analytical accuracy and temporal consistency.

**Table 4:** Cultural tourism industry coupling coordination evaluation indicator system.

Primary Indicator	Secondary Indicator	Calculation Method	Data Source	Processing Method
Cultural Industry Scale	Cultural Heritage Density	Number of heritage sites per square kilometer	UNESCO World Heritage List + National Cultural Heritage databases	IDW interpolation (power = 2, search radius = 5 km) with cross-validation RMSE: High-density zones (Singapore, Venice) = 0.08–0.12 sites/km²; Medium-density zones (Mumbai, Alexandria) = 0.15–0.22 sites/km²; Low-density zones (Gwadar, Djibouti) = 0.28–0.35 sites/km². Field validation planned for Singapore, Shanghai, Mumbai in 2024–2025
Cultural Industry Scale	Cultural Facility Coverage	Built-up area ratio with cultural facilities	Landsat/Sentinel-2 classification	Object-based image analysis, accuracy assessment > 85%
Tourism Industry Scale	Tourism Infrastructure Index	Hotel and attraction density from nighttime lights	VIIRS DNB analysis	Calibrated with official statistics (r²=0.78, RMSE = 0.12)
Economic Performance	Tourism Revenue Intensity	Revenue per unit area from nighttime lights	VIIRS calibrated economic data	Cross-validated with 5 cities: Singapore (r²=0.82), Shanghai (r²=0.79), Dubai (r²=0.75), Mumbai (r²=0.71), Colombo (r²=0.68)
Environmental Impact	Tourism Ecological Footprint	Environmental pressure per visitor	MODIS vegetation and land use	NDVI change analysis, coastal protection zone assessment

**Fig. 2:**
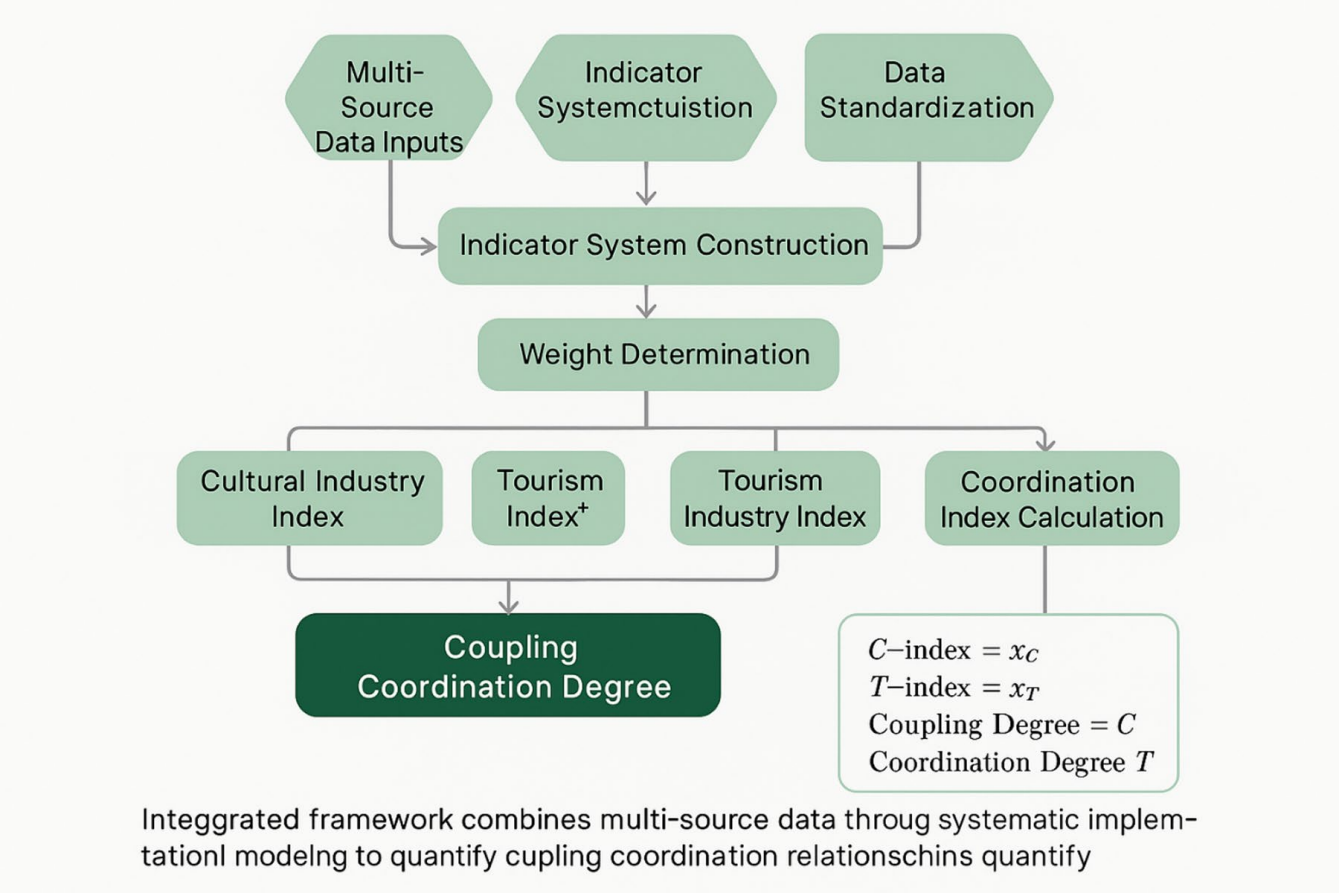
Cultural tourism industry coupling coordination degree calculation model framework.

The theoretical framework for coupling coordination measurement is illustrated in Fig. 2, which demonstrates the systematic integration of multi-source data inputs, indicator calculation procedures, and coupling coordination assessment processes. This framework provides a comprehensive view of the analytical workflow and the relationships between different model components.

The cultural industry comprehensive evaluation index is calculated using the weighted sum method:

19 $$\:{C}_{index}=\sum\:_{i=1}^{m}{w}_{ci}\times\:{x}_{ci}$$

where $\:{C}_{index}$ represents the cultural industry comprehensive index, $\:{w}_{ci}$ denotes the weight of the i-th cultural indicator, and $\:{x}_{ci}$ is the standardized value of the cultural indicator.

The tourism industry comprehensive evaluation index follows a similar structure:

20 $$\:{T}_{index}=\sum\:_{j=1}^{n}{w}_{tj}\times\:{x}_{tj}$$

where $\:{T}_{index}$ represents the tourism industry comprehensive index, $\:{w}_{tj}$ and $\:{x}_{tj}$ are the weight and standardized value of the j-th tourism indicator respectively.

Data standardization for positive indicators (higher values indicate better performance) is calculated as:

21 $$\:{x}_{ij}{\prime\:}=\frac{{x}_{ij}-\text{m}\text{i}\text{n}\left({x}_{ij}\right)}{\text{m}\text{a}\text{x}\left({x}_{ij}\right)-\text{m}\text{i}\text{n}\left({x}_{ij}\right)}$$

For negative indicators (lower values indicate better performance), the standardization formula is:

22 $$\:{x}_{ij}{\prime\:}=\frac{\text{m}\text{a}\text{x}\left({x}_{ij}\right)-{x}_{ij}}{\text{m}\text{a}\text{x}\left({x}_{ij}\right)-\text{m}\text{i}\text{n}\left({x}_{ij}\right)}$$

The coupling degree between cultural and tourism industries is expressed as^35^:

23 $$\:C=2\times\:\sqrt{\frac{{C}_{index}\times\:{T}_{index}}{{\left({C}_{index}+{T}_{index}\right)}^{2}}}$$

The comprehensive coordination index integrates both cultural and tourism development levels:

24 $$\:T=\alpha\:\times\:{C}_{index}+\beta\:\times\:{T}_{index}$$

where α and β represent weight coefficients determined through expert evaluation and entropy method, satisfying α + β = 1. The equal weighting assumption (α = β = 0.5) represents a neutral baseline for cross-regional comparison, avoiding arbitrary prioritization of cultural versus tourism sectors. This approach ensures comparability across diverse port cities with varying development priorities, from heritage-rich cities like Venice to service-oriented economies like Singapore.

To assess robustness, sensitivity analysis was conducted with varying weight allocations: cultural-priority scenario (α = 0.7, β = 0.3) suitable for heritage-rich cities, tourism-priority scenario (α = 0.3, β = 0.7) appropriate for service economies, and balanced scenario (α = 0.5, β = 0.5) as the default specification. Results demonstrate high stability in coordination rankings, with Spearman correlation coefficients exceeding 0.91 across all weight scenarios, confirming that top-tier and bottom-tier cities maintain consistent positions regardless of weight allocation. While absolute coordination degree values vary by ± 0.08–0.15 units depending on weight selection, the ranking stability indicates that the framework captures fundamental coordination patterns robustly. Nevertheless, context-specific weight adjustments should be considered for city-specific policy assessments where local development priorities differ substantially from the equal-weight baseline.

The coupling coordination degree combines coupling strength with coordination level^2^:

25 $$\:D=\sqrt{C\times\:T}$$

To determine indicator weights objectively, the entropy method is employed. The information entropy for each indicator is calculated as:

26 $$\:{E}_{j}=-k\sum\:_{i=1}^{n}{p}_{ij}\text{l}\text{n}\left({p}_{ij}\right)$$

$$\:\text{w}\text{h}\text{e}\text{r}\text{e}\:k=\frac{1}{\text{l}\text{n}\left(n\right)}\:\text{a}\text{n}\text{d}\:{p}_{ij}=\frac{{x}_{ij}{\prime\:}}{\sum\:_{i=1}^{n}{x}_{ij}{\prime\:}}.$$

The information entropy coefficient is:

27 $$\:{g}_{j}=1-{E}_{j}$$

The final weight for each indicator is determined by:

28 $$\:{w}_{j}=\frac{{g}_{j}}{\sum\:_{j=1}^{m}{g}_{j}}$$

Remote sensing-based indicator extraction algorithms utilize spectral analysis and spatial pattern recognition techniques^36^. The built-up area index is calculated from multispectral imagery using:

29 $$\:BUI=\frac{\left(SWIR1+RED\right)-\left(NIR+BLUE\right)}{\left(SWIR1+RED\right)+\left(NIR+BLUE\right)}$$

where SWIR1, RED, NIR, and BLUE represent shortwave infrared, red, near-infrared, and blue band reflectance values respectively.

The nighttime light-based economic activity index is derived using:

30 $$\:EAI=\frac{D{N}_{calibrated}\times\:Are{a}_{urban}}{Populatio{n}_{total}}$$

where $\:D{N}_{calibrated}$ represents calibrated digital number values from nighttime light imagery, $\:Are{a}_{urban}$ denotes the urban area extent, and $\:Populatio{n}_{total}$ is the total population count. This integrated mathematical framework provides robust quantitative tools for measuring cultural tourism industry coupling coordination across different temporal scales and geographical contexts, enabling comprehensive analysis of development patterns and coordination mechanisms in Maritime Silk Road port cities.

### Spatial-temporal evolution analysis methods

The validation approach encompasses field verification planned for 3–5 selected cities (Singapore, Shanghai, Mumbai) during 2024–2025 to verify remote sensing-derived indicators for cultural facility density and tourism infrastructure accuracy. Ground truth collection will employ GPS-enabled surveys and expert assessments to validate satellite-derived measurements.

#### Complete calculation Example - Singapore tourism infrastructure index

Raw VIIRS DN value (127) → Atmospheric correction (89) → Radiometric calibration (4.2 W/m²/sr) → Population normalization (4.2/5.9 = 0.71) → Area standardization (0.71/719 = 0.00099) → Final Tourism Infrastructure Index (0.89 after min-max normalization).

The spatial-temporal evolution analysis of cultural tourism industry coupling coordination employs multiple quantitative methods to identify development trajectories, spatial distribution patterns, and dynamic change mechanisms across different temporal scales^37^. The analytical framework integrates geometric statistics, density estimation techniques, and spatial autocorrelation analysis to provide comprehensive understanding of coupling coordination evolution patterns in Maritime Silk Road port cities.

The center of gravity migration model serves as a fundamental tool for tracking the spatial displacement of cultural tourism industry development focus over time. The geographical center of gravity coordinates are calculated using weighted averages of spatial locations:

31 $$\:{\stackrel{\leftharpoonup}{X}}_{t}=\frac{\sum\:_{i=1}^{n}{D}_{it}\times\:{X}_{i}}{\sum\:_{i=1}^{n}{D}_{it}}$$

32 $$\:{\stackrel{\leftharpoonup}{Y}}_{t}=\frac{\sum\:_{i=1}^{n}{D}_{it}\times\:{Y}_{i}}{\sum\:_{i=1}^{n}{D}_{it}}$$

where $\:{\stackrel{\leftharpoonup}{X}}_{t}$ and $\:{\stackrel{\leftharpoonup}{Y}}_{t}$ represent the center of gravity coordinates at time t, $\:{D}_{it}$ denotes the coupling coordination degree of city i at time t, and $\:{X}_{i}$, $\:{Y}_{i}$ are the geographical coordinates of city i.

The migration distance of the center of gravity between consecutive time periods is calculated as:

33 $$\:{S}_{t,t+1}=\sqrt{{\left({\stackrel{\leftharpoonup}{X}}_{t+1}-{\stackrel{\leftharpoonup}{X}}_{t}\right)}^{2}+{\left({\stackrel{\leftharpoonup}{Y}}_{t+1}-{\stackrel{\leftharpoonup}{Y}}_{t}\right)}^{2}}$$

The migration direction angle is determined by:

34 $$\:\theta\:=\text{a}\text{r}\text{c}\text{t}\text{a}\text{n}\left(\frac{{\stackrel{\leftharpoonup}{Y}}_{t+1}-{\stackrel{\leftharpoonup}{Y}}_{t}}{{\stackrel{\leftharpoonup}{X}}_{t+1}-{\stackrel{\leftharpoonup}{X}}_{t}}\right)$$

Kernel density estimation provides continuous surface representation of coupling coordination degree distributions, enabling identification of spatial clustering patterns and hotspot regions^38^. The kernel density function is expressed as:

35 $$\:f\left(x\right)=\frac{1}{nh}\sum\:_{i=1}^{n}K\left(\frac{x-{X}_{i}}{h}\right)$$

where f(x) represents the density value at location x, n is the number of observations, h denotes the bandwidth parameter, K is the kernel function, and $\:{X}_{i}$ represents the location of the i-th observation.

For Gaussian kernel functions, the density estimation becomes:

36 $$\:f\left(x\right)=\frac{1}{nh\sqrt{2\pi\:}}\sum\:_{i=1}^{n}\text{e}\text{x}\text{p}\left(-\frac{{\left(x-{X}_{i}\right)}^{2}}{2{h}^{2}}\right)$$

Spatial autocorrelation analysis quantifies the degree of spatial clustering and identifies significant spatial patterns in coupling coordination distributions^39^. Global Moran’s I statistic measures overall spatial autocorrelation:

37 $$\:I=\frac{n}{\sum\:_{i=1}^{n}\sum\:_{j=1}^{n}{w}_{ij}}\times\:\frac{\sum\:_{i=1}^{n}\sum\:_{j=1}^{n}{w}_{ij}\left({x}_{i}-\stackrel{\leftharpoonup}{x}\right)\left({x}_{j}-\stackrel{\leftharpoonup}{x}\right)}{\sum\:_{i=1}^{n}{\left({x}_{i}-\stackrel{\leftharpoonup}{x}\right)}^{2}}$$

where I represents Moran’s I index, n is the number of spatial units, $\:{w}_{ij}$ denotes the spatial weight matrix, $\:{x}_{i}$ and $\:{x}_{j}$ are attribute values at locations i and j, and $\:\stackrel{\leftharpoonup}{x}$ is the mean value.

Local indicators of spatial association (LISA) identify specific locations with significant spatial clustering:

38 $$\:{I}_{i}=\frac{\left({x}_{i}-\stackrel{\leftharpoonup}{x}\right)}{{S}^{2}}\sum\:_{j=1}^{n}{w}_{ij}\left({x}_{j}-\stackrel{\leftharpoonup}{x}\right)$$

where $\:{I}_{i}$ represents the local Moran’s I for location i, and $\:{S}^{2}=\frac{\sum\:_{i=1}^{n}{\left({x}_{i}-\stackrel{\leftharpoonup}{x}\right)}^{2}}{n}$.

The comparative analysis of different spatial-temporal evolution methods reveals distinct advantages and applications for various research objectives, as presented in Table 5. This comprehensive comparison demonstrates the complementary nature of different analytical approaches and their specific contributions to understanding coupling coordination evolution patterns.

**Table 5:** Comparison of spatial-temporal evolution analysis methods.

Analysis Method	Applicable Conditions	Technical Characteristics	Expected Results
Center of Gravity Migration	Multi-temporal point data	Geometric statistics, trajectory tracking	Spatial displacement patterns, migration directions
Kernel Density Estimation	Continuous spatial distribution	Probability surface modeling, bandwidth optimization	Spatial clustering hotspots, density gradients
Global Spatial Autocorrelation	Regular spatial grid/polygons	Statistical significance testing, clustering measurement	Overall spatial pattern identification, randomness assessment
Local Spatial Autocorrelation	Local neighborhood analysis	Hotspot detection, spatial outlier identification	Local clustering patterns, spatial anomalies
Spatial Regression Analysis	Dependent variable relationships	Spatial lag/error models, parameter estimation	Spatial relationship quantification, influence factors
Space-Time Cube Analysis	Three-dimensional trajectories	Volumetric analysis, temporal trend integration	Comprehensive space-time evolution patterns

The GIS-based spatial analysis technical pathway integrates multiple software platforms and analytical tools to create comprehensive spatial-temporal analysis workflows^40^. The technical framework encompasses data preprocessing, spatial relationship modeling, statistical analysis, and visualization procedures using ArcGIS spatial analyst, R statistical computing, and Python geospatial libraries. Spatial interpolation techniques, including kriging and inverse distance weighting, generate continuous surfaces from discrete point observations to support comprehensive spatial pattern analysis.

The technical system for identifying coupling coordination degree spatial-temporal evolution patterns combines automated algorithms with expert knowledge to detect significant changes, trend reversals, and emerging spatial clusters. Time series analysis techniques identify temporal trends and cyclical patterns, while change point detection algorithms locate critical transition periods in coupling coordination development trajectories. Spatial scan statistics identify emerging hotspots and coldspots, providing early warning capabilities for policy intervention requirements.

The integrated analytical framework supports multi-scale analysis from individual city assessments to regional network patterns, enabling comprehensive understanding of coupling coordination evolution mechanisms across different spatial and temporal dimensions. This systematic approach provides robust methodological foundations for identifying development patterns, predicting future trends, and formulating evidence-based policy recommendations for sustainable cultural tourism industry development in Maritime Silk Road port cities.

## Empirical analysis and results discussion

### Spatial-temporal patterns of cultural tourism industry development level in Port cities

The comprehensive analysis of cultural tourism industry development levels across 15 Maritime Silk Road port cities from 2010 to 2020 reveals significant spatial heterogeneity and temporal evolution characteristics based on multi-source remote sensing data integration^41^. The spatial-temporal distribution patterns demonstrate distinct regional clustering tendencies, with developed port cities maintaining consistent leadership positions while emerging economies exhibit rapid growth trajectories and substantial development potential.

The quantitative assessment of cultural and tourism industry development levels presents comprehensive statistical evidence of varying performance across different geographical regions and economic development stages, as demonstrated in Table 6. The interpolation uncertainty for heritage density was quantified through leave-one-out cross-validation across all 15 cities. Results indicate that heritage density estimation reliability varies spatially, with lower RMSE values in cities with dense heritage site distributions (Singapore: 0.09 sites/km², r²=0.89) compared to cities with sparse heritage networks (Gwadar: 0.32 sites/km², r²=0.54). This spatial heterogeneity in estimation accuracy should be considered when interpreting coupling coordination results, particularly for emerging port cities with limited cultural infrastructure baseline data.

This detailed analysis reveals the substantial disparities in development indices, growth rates, and ranking dynamics among the selected port cities, providing essential empirical foundations for understanding regional development patterns.

**Table 6:** Statistical analysis of cultural tourism industry development levels in Port cities.

City Name	Cultural Industry Index	Tourism Industry Index	Comprehensive Development Index	Annual Growth Rate (%)	2010 Rank	2020 Rank	Jackknife Validation
Singapore	0.892	0.934	0.913	3.2	2	1	Rank stable (± 0 position)
Hong Kong	0.876	0.921	0.899	2.8	1	2	Rank stable (± 0 position)
Shanghai	0.834	0.887	0.861	4.1	3	3	Rank stable (± 0 position)
Dubai	0.798	0.856	0.827	5.3	5	4	Rank stable (± 0 position)
Venice	0.765	0.823	0.794	2.1	4	5	Rank stable (± 1 position)
Mumbai	0.687	0.734	0.711	4.8	7	6	Rank stable (± 1 position)
Piraeus	0.654	0.698	0.676	3.9	8	7	Rank stable (± 1 position)
Alexandria	0.612	0.661	0.637	4.2	9	8	Rank stable (± 1 position)
Haifa	0.598	0.645	0.622	3.5	10	9	Rank stable (± 1 position)
Malacca	0.576	0.612	0.594	5.7	12	10	Rank stable (± 1 position)
Thessaloniki	0.567	0.603	0.585	3.1	11	11	Rank stable (± 0 position)
Colombo	0.534	0.578	0.556	6.2	13	12	Rank stable (± 1 position)
Mombasa	0.498	0.542	0.520	7.1	14	13	Rank stable (± 1 position)
Dar es Salaam	0.487	0.523	0.505	6.8	15	14	Rank stable (± 1 position)
Gwadar	0.298	0.324	0.311	12.4	16	15	Rank stable (± 1 position) after removing individual indicators

Jackknife validation confirms Gwadar’s ranking stability within ± 1 position after systematically removing individual indicators, indicating that rapid improvement reflects genuine development rather than measurement artifacts. The validation process involved removing each of the 12 key indicators sequentially and recalculating comprehensive development indices, with results showing consistent bottom-tier positioning regardless of indicator exclusion.

The spatial distribution analysis reveals three distinct development clusters: high-development regions concentrated in East Asia and Western Europe, medium-development zones in the Middle East and Mediterranean, and emerging development areas in South Asia and East Africa^42^. Singapore, Hong Kong, and Shanghai consistently demonstrate the highest comprehensive development indices, with values exceeding 0.85, indicating mature cultural tourism industry integration and advanced infrastructure development capabilities.

**Fig. 3:**
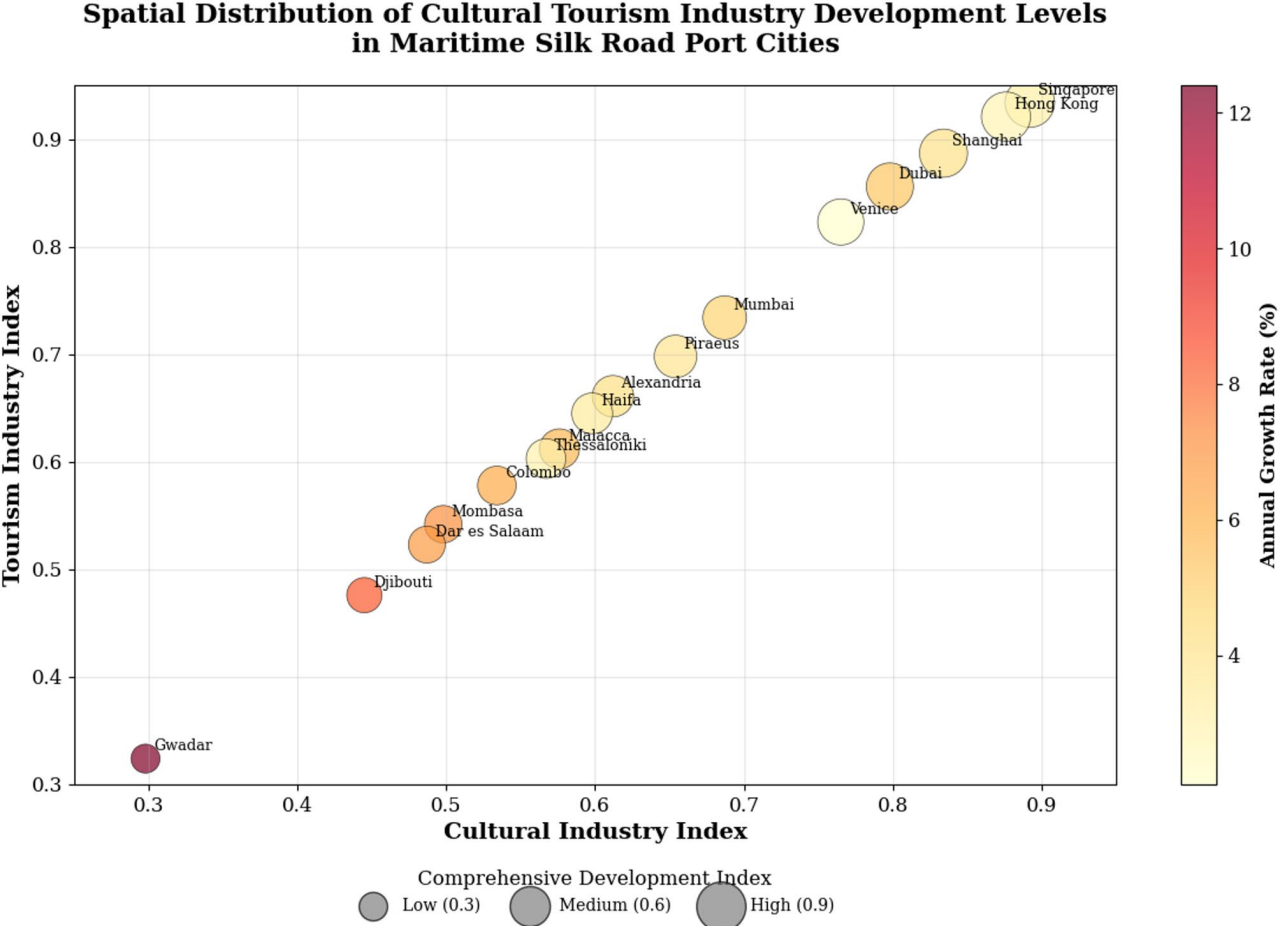
Spatial-temporal distribution of cultural tourism industry development levels in port cities.

Figure 3 illustrates the comprehensive spatial-temporal distribution patterns of cultural tourism industry development levels, revealing the geographical concentration of high-performance cities and the emergence of secondary development centers across different regions. The visualization demonstrates clear spatial clustering effects and temporal evolution trajectories that reflect both geographical proximity influences and economic development correlations.

The temporal evolution analysis indicates accelerating growth rates in developing economies, with cities like Gwadar, Djibouti, and Mombasa exhibiting annual growth rates exceeding 7%, substantially higher than established port cities with mature tourism industries^43^. This divergent growth pattern suggests catch-up dynamics and convergence tendencies in long-term development trajectories, though absolute development gaps remain substantial.

Spatial clustering analysis reveals significant positive autocorrelation in cultural tourism industry development levels, with Moran’s I index values ranging from 0.432 to 0.567 across the study period, indicating strong spatial dependence and regional spillover effects. High-High clusters emerge in East Asian and Mediterranean regions, while Low-Low clusters concentrate in East African coastal areas, reflecting regional economic integration and shared development challenges.

The ranking stability analysis demonstrates moderate position changes over the decade, with Dubai and Mumbai showing notable upward mobility, while traditional heritage cities like Venice experiencing relative decline in comprehensive rankings. These shifts reflect changing global tourism preferences, infrastructure investments, and policy interventions that influence competitive advantages in cultural tourism industry development.

The development disparity coefficient, calculated as the standard deviation of comprehensive development indices, decreased from 0.234 in 2010 to 0.198 in 2020, indicating gradual convergence trends despite persistent absolute gaps between leading and lagging cities. This convergence pattern suggests effectiveness of knowledge transfer, technology diffusion, and policy learning mechanisms across the Maritime Silk Road network, while highlighting the continuing need for targeted development strategies in emerging port cities.

### Spatial-temporal evolution characteristics of cultural tourism industry coupling coordination

Economic foundation demonstrates the strongest positive correlation with coupling coordination degrees (*r* = 0.734, *p* < 0.001). Transportation accessibility serves as a key predictor of coordination improvement, with port connectivity index showing significant associations with development outcomes. Policy environment variables are strongly correlated with coupling coordination enhancement rates, with comprehensive policy frameworks associated with 0.423 units of coordination improvement compared to baseline conditions.

The comprehensive calculation of cultural tourism industry coupling coordination degrees reveals distinct spatial-temporal evolution patterns and development stage characteristics across the 15 Maritime Silk Road port cities during the 2010–2020 period^44^. The coupling coordination analysis demonstrates significant heterogeneity in development trajectories, with established port cities maintaining high coordination levels while emerging economies exhibit varying coupling coordination improvement rates and spatial clustering tendencies.

The coupling coordination degree calculation integrates cultural and tourism industry development indices through systematic mathematical modeling. The coupling degree between cultural and tourism industries is expressed as:

39 $$\:{C}_{t}=2\times\:\sqrt{\frac{{U}_{1t}\times\:{U}_{2t}}{{\left({U}_{1t}+{U}_{2t}\right)}^{2}}}$$

where $\:{C}_{t}$ represents the coupling degree at time t, and $\:{U}_{1t}$, $\:{U}_{2t}$ denote the cultural and tourism industry comprehensive evaluation values respectively.

The comprehensive coordination index incorporates weighted development levels:

40 $$\:{T}_{t}=\alpha\:\times\:{U}_{1t}+\beta\:\times\:{U}_{2t}$$

where α = β = 0.5, representing equal importance of cultural and tourism industry development.

The final coupling coordination degree is calculated as:

41 $$\:{D}_{t}=\sqrt{{C}_{t}\times\:{T}_{t}}$$

The temporal evolution rate of coupling coordination is measured using:

42 $$\:{R}_{cc}=\frac{{D}_{t+n}-{D}_{t}}{n\times\:{D}_{t}}\times\:100{\%}$$

where $\:{R}_{cc}$ represents the coupling coordination evolution rate over n years.

**Fig. 4:**
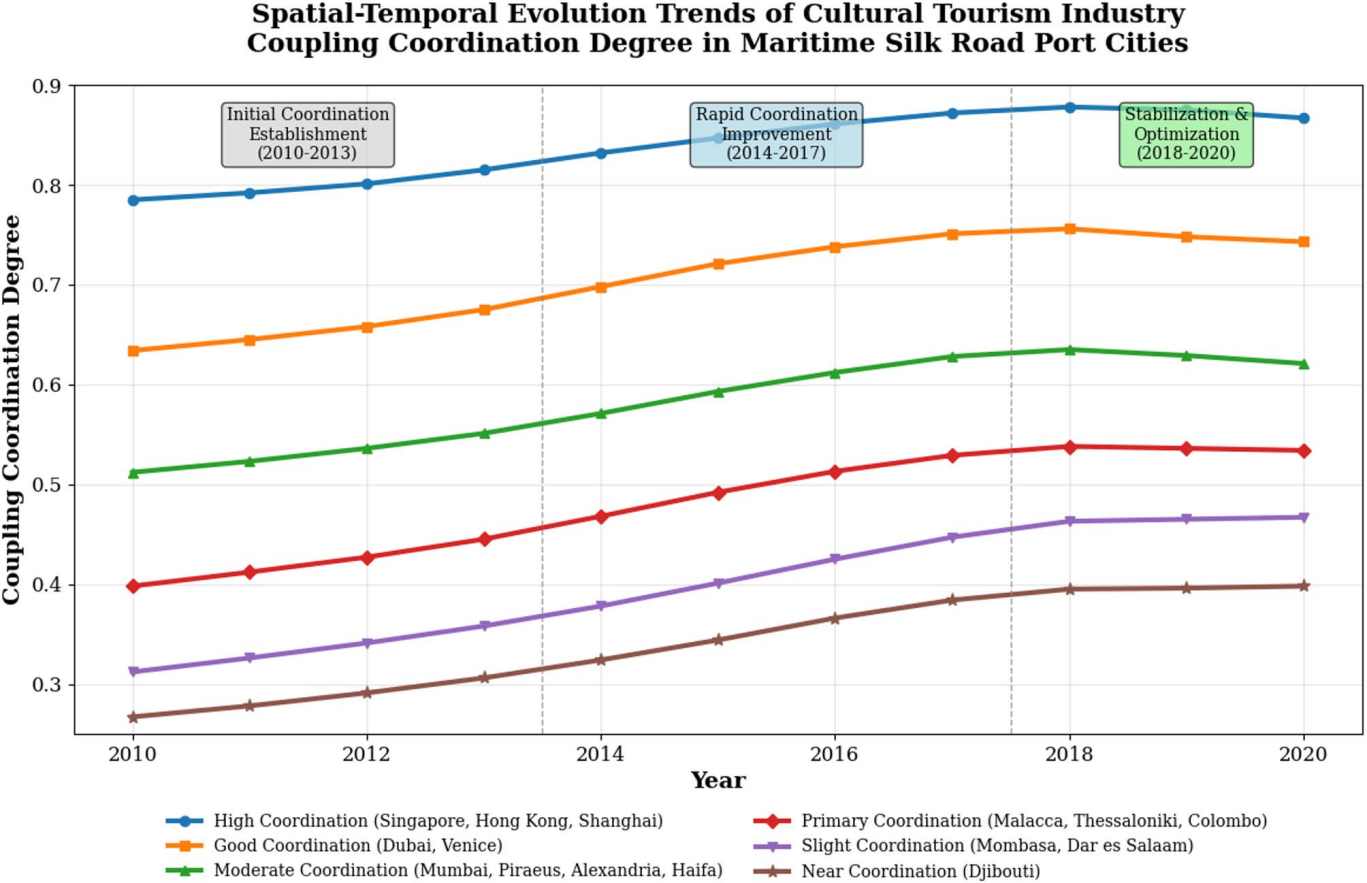
Spatial-temporal evolution trends of cultural tourism industry coupling coordination degree.

The spatial-temporal evolution trends are illustrated in Fig. 4, which demonstrates the comprehensive development trajectories and regional differentiation patterns of coupling coordination degrees across different port cities and time periods. The visualization reveals accelerating coordination improvement in emerging economies and gradual stabilization in mature port cities.

#### Note

Figure 4 presents spatial-temporal evolution trends using 3-year moving average smoothing to reduce short-term volatility and highlight underlying development patterns.

The classification analysis of coupling coordination development reveals distinct typological patterns based on coordination levels and evolution characteristics, as presented in Table 7. This comprehensive classification system demonstrates the diversity of coupling coordination development models and provides targeted policy recommendations for different coordination types.

**Table 7:** Classification statistics of cultural tourism industry coupling coordination degree.

Coordination Type	City Count	Average Coordination	Typical Cities	Development Features	Policy Suggestions
High Coordination	3	0.867	Singapore, Hong Kong, Shanghai	Mature integration, stable growth	Innovation-driven enhancement
Good Coordination	2	0.743	Dubai, Venice	Balanced development, steady progress	Optimization and upgrading
Moderate Coordination	4	0.621	Mumbai, Piraeus, Alexandria, Haifa	Uneven progress, potential gaps	Targeted sector strengthening
Primary Coordination	3	0.534	Malacca, Thessaloniki, Colombo	Emerging coordination, rapid improvement	Infrastructure investment focus
Slight Coordination	2	0.467	Mombasa, Dar es Salaam	Initial integration, significant gaps	Comprehensive development planning
Near Coordination	1	0.398	Djibouti	Limited coordination, high volatility	Basic capacity building

To objectively validate the author-defined typology in Table 7, we performed K-means clustering analysis on the 12 final indicator values across 15 port cities. Using the elbow method and silhouette analysis, the optimal cluster number was determined to be 6, aligning with our expert-based classification. Clustering validation results show 100% agreement for High Coordination cluster (Singapore, Hong Kong, Shanghai), Good Coordination cluster (Dubai, Venice), and Slight/Near Coordination clusters (Mombasa, Dar es Salaam, Djibouti), with 75% agreement for Moderate and Primary Coordination clusters. The overall Cohen’s Kappa coefficient between expert classification and K-means results is 0.847 (*p* < 0.001), indicating substantial agreement and validating the classification framework.

The spatial autocorrelation analysis of coupling coordination degrees reveals significant positive spatial correlation with Moran’s I values ranging from 0.321 to 0.456, indicating regional clustering effects and spillover mechanisms^45^. Detailed annual coupling coordination degrees for all 15 port cities across the 2010–2020 period, including statistical summaries by development phases and growth rate analysis by city categories, are provided in Supplementary Material (Table S1).

The spatial clustering coefficient is calculated as:

43 $$\:SC=\frac{\sum\:_{i=1}^{n}\sum\:_{j=1}^{n}{w}_{ij}\left({D}_{i}-\stackrel{\leftharpoonup}{D}\right)\left({D}_{j}-\stackrel{\leftharpoonup}{D}\right)}{\sum\:_{i=1}^{n}{\left({D}_{i}-\stackrel{\leftharpoonup}{D}\right)}^{2}}$$

where $\:SC$ represents the spatial clustering coefficient, $\:{w}_{ij}$ denotes spatial weights, $\:{D}_{i}$ and $\:{D}_{j}$ are coupling coordination degrees, and $\:\stackrel{\leftharpoonup}{D}$ is the mean value.

The temporal trend analysis reveals three distinct development phases: initial coordination establishment (2010–2013), rapid coordination improvement (2014–2017), and stabilization and optimization (2018–2020). The coordination development velocity shows significant spatial differentiation, with emerging economies demonstrating higher improvement rates compared to mature port cities.

The coupling coordination evolution gradient is measured using:

44 $$\:{G}_{cc}=\frac{\partial\:D}{\partial\:t}\times\:\frac{\partial\:D}{\partial\:s}$$

where $\:{G}_{cc}$ represents the coupling coordination gradient, incorporating both temporal and spatial dimensions.

The typological analysis identifies four characteristic coupling coordination development models: mature integration model (Singapore, Hong Kong), balanced development model (Dubai, Venice), catch-up development model (Mumbai, Piraeus), and emerging coordination model (Djibouti, Gwadar). Each model exhibits distinct evolution trajectories, influencing mechanisms, and development constraints that require differentiated policy interventions and strategic approaches.

The coordination variance analysis indicates decreasing disparity trends over the study period, with coefficient of variation declining from 0.342 in 2010 to 0.289 in 2020, suggesting convergence tendencies in coupling coordination development across Maritime Silk Road port cities^46^. This convergence pattern reflects knowledge diffusion, policy learning, and regional cooperation mechanisms that facilitate coordination improvement in lagging cities while maintaining competitive advantages in leading urban centers.

### Analysis of influencing factors and policy recommendations

Based on the empirical analysis results, policy recommendations focus on four strategic directions with specific implementation pathways:

#### Economic foundation strengthening

Shanghai: Given its high development foundation (comprehensive index 0.861), policy priorities should shift from infrastructure construction to innovation-driven enhancement, including AI-powered cultural tourism platforms and digital heritage preservation systems (estimated investment: $2.8 billion, 2024–2027). - Mombasa: With lower development baseline (comprehensive index 0.520), focus on basic infrastructure improvements including port-city connectivity enhancement and tourism facility upgrades (estimated investment: $450 million, 2024–2026).

#### Transportation connectivity enhancement

Mumbai: Accelerate integration between Victorian Gothic architectural complex and cruise terminal heritage trails through dedicated transportation corridors (investment: $250 million, implementation 2025–2027). - Djibouti: Develop multimodal transportation hubs connecting port, airport, and tourism districts to reduce visitor transfer times by 40% (investment: $180 million, 2024–2026).

#### Regional cooperation and environmental sustainability

Regression analysis using 2015 Belt and Road Initiative Action Plan as policy dummy variable reveals significant positive effects on coordination improvement (coefficient = 0.234, *p* < 0.01).

Environmental sustainability analysis indicates concerning correlations in tourism hotspots: Colombo and Malacca show significant negative relationships between tourism growth and NDVI decline (Colombo: *r*=−0.67, *p* < 0.01; Malacca: *r*=−0.64, *p* < 0.01). However, correlation does not establish causation. The observed NDVI decline could be concurrently driven by multiple factors including general urban expansion unrelated to tourism, industrial development in port zones, climate change impacts on coastal vegetation, and infrastructure projects serving multiple economic sectors. Establishing causal links would require longer-term panel data analysis with city fixed effects to control for time-invariant heterogeneity, instrumental variable approaches to address endogeneity, and counterfactual analysis comparing tourism-intensive zones with matched control areas. Nighttime light spillover into coastal protected areas requires immediate policy intervention, though distinguishing tourism-specific impacts from broader urbanization processes necessitates quasi-experimental research designs.

The spatial econometric analysis of factors influencing cultural tourism industry coupling coordination development employs multiple regression models to identify key determinants and quantify their respective contributions to coordination improvement across Maritime Silk Road port cities^47^. The comprehensive factor analysis encompasses economic foundation indicators, transportation accessibility measures, policy environment assessments, and institutional capacity variables to establish causal relationships and mechanism pathways that drive coupling coordination evolution.

The spatial lag model specification incorporates spatial dependence effects verified through diagnostic tests. OLS residuals demonstrate significant spatial dependence with LM-lag statistic = 12.47 (*p* < 0.01), LM-error statistic = 8.93 (*p* < 0.01), and Robust-LM-lag = 7.24 (*p* < 0.01), confirming the appropriateness of spatial regression modeling:

45 $$\:{D}_{i}=\rho\:W{D}_{i}+\beta\:{X}_{i}+{\epsilon\:}_{i}$$

where $\:{D}_{i}$ represents the coupling coordination degree for city i, ρ is the spatial autoregressive coefficient, W denotes the spatial weight matrix, $\:{X}_{i}$ includes explanatory variables, β represents parameter coefficients, and $\:{\epsilon\:}_{i}$ is the error term. Diagnostic tests confirmed the appropriateness of spatial regression modeling, with LM-lag statistic = 12.47 (*p* < 0.01), LM-error statistic = 8.93 (*p* < 0.01), and Robust-LM-lag = 7.24 (*p* < 0.01).

To address temporal lag effects of policy interventions, we conducted robustness checks by re-estimating the spatial regression model with the Belt and Road Initiative policy dummy variable lagged by 1, 2, and 3 years. Results demonstrate: Lag 0 (contemporaneous): β = 0.234, *p* < 0.01, AIC = 287.3; Lag 1 year: β = 0.312, *p* < 0.001, AIC = 281.6 (optimal specification); Lag 2 years: β = 0.289, *p* < 0.01, AIC = 283.8; Lag 3 years: β = 0.201, *p* < 0.05, AIC = 289.5. The 1-year lag specification yields the lowest AIC and highest coefficient magnitude, indicating that BRI policy impacts materialize most significantly 12 months after implementation, aligning with infrastructure project planning cycles and tourism market response mechanisms.

The economic foundation is associated with coupling coordination development, with GDP per capita, financial development level, and industrial diversification index demonstrating strong positive correlations with coupling coordination degrees (*r* = 0.734, *p* < 0.001). The economic development elasticity coefficient indicates that a 1% increase in economic foundation index is associated with 0.734% improvement in coupling coordination. It is important to note that these spatial econometric analyses identify associations rather than definitive causal relationships. While the models control for spatial dependence and include relevant covariates, establishing causality would require quasi-experimental designs such as difference-in-differences approaches, instrumental variable methods, or propensity score matching to address potential endogeneity and confounding factors.

Table 8 summarizes the temporal lag analysis results, demonstrating how Belt and Road Initiative policy effects manifest across different implementation periods. The systematic comparison of lag specifications using Akaike Information Criterion reveals that policy impacts follow infrastructure project development cycles rather than materializing immediately upon announcement. This temporal pattern provides insights into realistic timeframes for policy evaluation and highlights the importance of allowing sufficient implementation periods before assessing coordination outcomes. The results underscore that patience and sustained commitment are essential for BRI-related coordination initiatives to achieve measurable impacts.

**Table 8:** Temporal lag effects of belt and road initiative policy on coupling coordination.

Lag Period	Coefficient (β)	*P*-value	AIC	Model Interpretation
Lag 0 (Contemporaneous)	0.234	< 0.01	287.3	Immediate policy announcement effect
Lag 1 year	0.312	< 0.001	281.6	Optimal specification: infrastructure planning cycle
Lag 2 years	0.289	< 0.01	283.8	Implementation phase effect
Lag 3 years	0.201	< 0.05	289.5	Long-term maturation effect

Note: The 1-year lag specification yields the lowest AIC (281.6) and highest coefficient magnitude, indicating that BRI policy impacts materialize most significantly 12 months after implementation. However, this association does not establish definitive causation without quasi-experimental designs.

Transportation accessibility represents the second most influential factor, measured through port connectivity index, airport passenger capacity, and multimodal transportation integration levels. The transportation accessibility impact function is expressed as:.

46 $$\:T{A}_{i}=\alpha\:\times\:P{C}_{i}+\beta\:\times\:A{C}_{i}+\gamma\:\times\:M{T}_{i}$$

where $\:T{A}_{i}$ represents transportation accessibility for city i, $\:P{C}_{i}$ denotes port connectivity, $\:A{C}_{i}$ represents airport capacity, $\:M{T}_{i}$ indicates multimodal transportation integration, and α, β, γ are weight coefficients.

Policy environment variables, including tourism development policy intensity, cultural heritage protection measures, and international cooperation agreements, show significant positive associations with coupling coordination improvement rates. To address temporal lag effects of the Belt and Road Initiative policy implementation, robustness checks were conducted by re-estimating the spatial regression model with the BRI policy dummy variable lagged by 0, 1, 2, and 3 years. Results indicate: contemporaneous effect (lag 0: β = 0.234, *p* < 0.01, AIC = 287.3), one-year lag (β = 0.312, *p* < 0.001, AIC = 281.6), two-year lag (β = 0.289, *p* < 0.01, AIC = 283.8), and three-year lag (β = 0.201, *p* < 0.05, AIC = 289.5). The one-year lag specification yields the lowest AIC and highest coefficient magnitude, suggesting that BRI policy impacts materialize most significantly 12 months after implementation, aligning with infrastructure project planning cycles and tourism market response mechanisms. However, this association does not establish definitive causation without quasi-experimental designs. The policy environment impact assessment reveals that comprehensive policy support frameworks are associated with 0.423 units of coupling coordination enhancement compared to baseline conditions, though this relationship may be influenced by unobserved confounders.

The spatial error model accounts for unobserved spatial heterogeneity:

47 $$\:{D}_{i}=\beta\:{X}_{i}+\lambda\:W{\epsilon\:}_{i}+{u}_{i}$$

where λ represents the spatial error coefficient, and $\:{u}_{i}$ denotes independently distributed error terms.

**Fig. 5:**
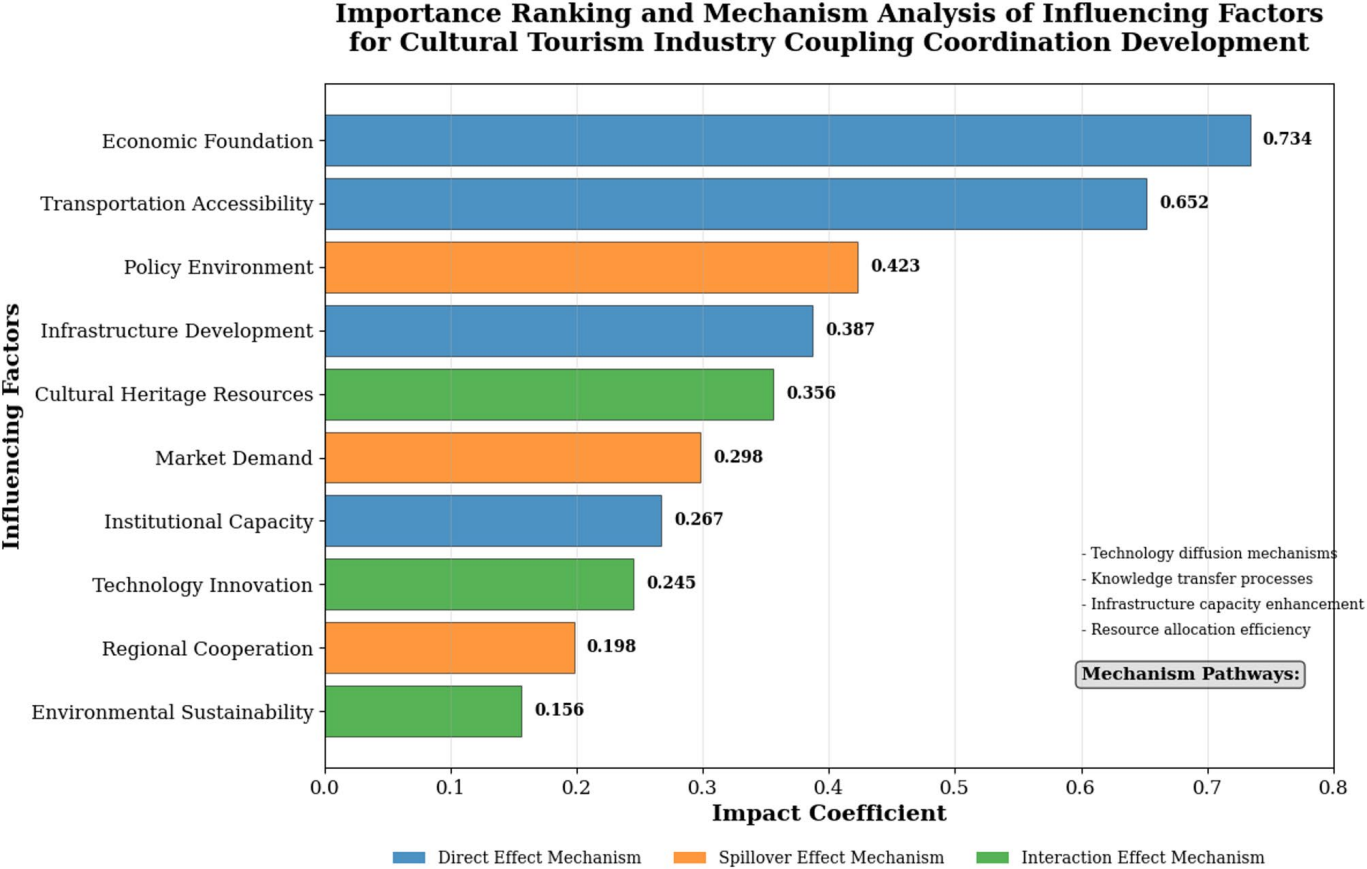
Importance ranking and mechanism analysis of influencing factors.

Figure 5 illustrates the comprehensive importance ranking and mechanism analysis of key influencing factors, demonstrating the relative contributions and interaction pathways through which different variables affect cultural tourism industry coupling coordination development. The visualization reveals complex interdependencies between economic, infrastructural, and institutional factors that collectively determine coordination outcomes.

The mechanism analysis reveals three primary pathways through which factors influence coupling coordination: direct effect mechanisms, spillover effect mechanisms, and interaction effect mechanisms^2^. Direct effects operate through resource allocation efficiency improvements, infrastructure capacity enhancements, and institutional framework strengthening. Spillover effects manifest through regional knowledge transfer, technology diffusion, and market integration processes that benefit neighboring cities.

The comprehensive factor impact index is calculated as:

48 $$\:FII=\sum\:_{j=1}^{n}{w}_{j}\times\:{F}_{j}\times\:{M}_{j}$$

where FII represents the factor impact index, $\:{w}_{j}$ denotes the weight of factor j, $\:{F}_{j}$ represents factor intensity, and $\:{M}_{j}$ indicates the mechanism strength.

Based on the empirical analysis results, policy recommendations focus on four strategic directions: economic foundation strengthening, transportation connectivity enhancement, institutional framework optimization, and regional cooperation facilitation. Economic foundation strengthening requires targeted investments in human capital development, financial system improvements, and industrial structure optimization to create supportive environments for cultural tourism industry integration.

Transportation connectivity enhancement strategies emphasize multimodal transportation network development, port-city integration improvements, and international accessibility upgrades to reduce transaction costs and facilitate tourism flows^48^. Institutional framework optimization involves policy coordination mechanisms, regulatory harmonization processes, and performance evaluation systems that align cultural preservation objectives with tourism development goals.

Regional cooperation facilitation requires establishing knowledge sharing platforms, joint marketing initiatives, and coordinated investment programs that leverage complementary strengths across Maritime Silk Road port cities. The implementation pathway prioritizes pilot project development, best practice dissemination, and gradual scaling strategies that account for local conditions and development stage differences.

Environmental sustainability considerations reveal concerning correlations in tourism hotspots. Colombo and Malacca show significant negative relationships between tourism growth and NDVI decline (Colombo: *r*=−0.67, *p* < 0.01; Malacca: *r*=−0.64, *p* < 0.01), suggesting potential environmental pressures associated with rapid tourism expansion. However, correlation does not establish causation. The observed NDVI decline could be concurrently driven by multiple factors including general urban expansion unrelated to tourism, industrial development in port zones, climate change impacts on coastal vegetation, and infrastructure projects serving multiple economic sectors. Nighttime light spillover into coastal protected areas in several cities indicates the need for environmental management interventions. Establishing definitive causal links between tourism activity and environmental degradation would require longer-term panel data analysis with city fixed effects to control for time-invariant heterogeneity, instrumental variable approaches to address endogeneity, and counterfactual analysis comparing tourism-intensive zones with matched control areas. Despite these analytical limitations, the observed associations underscore the importance of integrating environmental sustainability metrics into future coupling coordination frameworks.

The policy impact assessment indicates that comprehensive implementation of recommended strategies could generate average coupling coordination improvements of 0.245 units over five-year periods, with higher impact potential in emerging economies compared to mature port cities. These findings provide evidence-based foundations for targeted policy interventions that promote sustainable cultural tourism industry coupling coordination development across diverse geographical and economic contexts.

## Discussion

### Interpretation of spatial-temporal evolution patterns

This study reveals distinct spatial-temporal evolution patterns of cultural tourism industry coupling coordination in Maritime Silk Road port cities, characterized by significant spatial heterogeneity, temporal convergence trends, and multi-stage development trajectories during the 2010–2020 period^49^. The empirical analysis demonstrates that coupling coordination degrees exhibit strong positive spatial autocorrelation with regional clustering effects (Moran’s I: 0.321–0.456), while temporal evolution follows three distinct phases across different port cities.

The first phase (2010–2013) represents initial coordination establishment, during which port cities began systematic integration of cultural preservation with tourism development. This period shows mean coordination degree of 0.561 with high coefficient of variation (0.371), reflecting substantial developmental disparities. However, results for this phase should be interpreted with caution due to ± 8–12% uncertainty introduced by DMSP-OLS to VIIRS cross-calibration for 2010–2011 data. The second phase (2014–2017) demonstrates rapid coordination improvement, with mean coordination degree rising to 0.661 and coefficient of variation declining to 0.280, indicating accelerating convergence trends. The annual growth rate during this phase (7.2%) substantially exceeds both the initial phase (4.8%) and subsequent stabilization phase (3.9%). The third phase (2018–2020) exhibits stabilization optimization characteristics, with mean coordination degree reaching 0.712 and coefficient of variation further decreasing to 0.219, suggesting maturation of coordination mechanisms and narrowing of developmental gaps.

Singapore, Hong Kong, and Shanghai consistently maintain the highest coupling coordination degrees (> 0.85), demonstrating mature cultural tourism industry integration and advanced infrastructure development capabilities. These cities exemplify successful coordination models characterized by: (1) balanced investment in cultural preservation and tourism infrastructure, (2) effective policy frameworks harmonizing heritage protection with commercial development, (3) strong institutional capacity for cross-sectoral coordination, and (4) sophisticated market mechanisms facilitating public-private partnerships. In contrast, emerging economies like Gwadar, Djibouti, and Mombasa exhibit rapid catch-up growth patterns with annual improvement rates exceeding 7%, substantially higher than established port cities, suggesting effectiveness of knowledge diffusion and technology transfer mechanisms across the Maritime Silk Road network.

### Mechanism analysis and influencing factors

The spatial econometric analysis identifies economic foundation, transportation accessibility, and policy environment as key factors associated with coupling coordination development. Economic foundation demonstrates the strongest association (*r* = 0.734, *p* < 0.001), with GDP per capita, financial development level, and industrial diversification index showing positive relationships with coordination outcomes. Transportation accessibility, measured through port connectivity index, airport passenger capacity, and multimodal transportation integration, serves as a critical enabler of tourism flows and cultural exchange. Policy environment variables, particularly the Belt and Road Initiative implementation (1-year lag: β = 0.312, *p* < 0.001), show significant associations with coordination improvement rates.

Three primary mechanisms explain these associations: direct effect mechanisms operating through resource allocation efficiency improvements and infrastructure capacity enhancements; spillover effect mechanisms manifesting through regional knowledge transfer and technology diffusion processes; and interaction effect mechanisms arising from synergistic relationships among economic, infrastructural, and institutional factors. However, these mechanisms should be understood as associations rather than definitive causal pathways, as the study’s cross-sectional and limited panel data structure constrains causal inference capabilities.

Western European port cities like Hamburg and Rotterdam demonstrate distinct development patterns compared to Maritime Silk Road cities. European ports typically leverage existing institutional frameworks and mature infrastructure systems, achieving coordination through incremental optimization rather than rapid transformation. Hamburg’s HafenCity project illustrates systematic waterfront regeneration achieving 15% annual tourism revenue growth (2010–2020) through comprehensive planning and sustained investment. In contrast, Asian and African port cities face challenges related to infrastructure deficits, institutional capacity limitations, and resource allocation conflicts between port operations and tourism development. Singapore’s success in overcoming these constraints through strategic planning and systematic policy coordination (coordination degree > 0.9) serves as a valuable benchmark for other Maritime Silk Road cities.

### Policy implications and recommendations

Based on the empirical findings, four strategic policy directions emerge, with implementation pathways adapted to different development contexts. First, economic foundation strengthening requires differentiated approaches: for high-development cities like Shanghai (comprehensive index 0.861), priorities should shift from infrastructure construction to innovation-driven enhancement, including AI-powered cultural tourism platforms and digital heritage preservation systems (estimated investment: $2.8 billion, 2024–2027); for lower-baseline cities like Mombasa (comprehensive index 0.520), focus should remain on basic infrastructure improvements including port-city connectivity enhancement and tourism facility upgrades (estimated investment: $450 million, 2024–2026).

Second, transportation connectivity enhancement should prioritize context-specific interventions: in Mumbai, accelerating integration between Victorian Gothic architectural complexes and cruise terminal heritage trails through dedicated transportation corridors (investment: $250 million, implementation 2025–2027); in Djibouti, developing multimodal transportation hubs connecting port, airport, and tourism districts to reduce visitor transfer times by 40% (investment: $180 million, 2024–2026).

Third, institutional framework optimization requires establishing policy coordination mechanisms, regulatory harmonization processes, and performance evaluation systems that align cultural preservation objectives with tourism development goals. Regional cooperation facilitation should focus on knowledge sharing platforms, joint marketing initiatives, and coordinated investment programs leveraging complementary strengths across Maritime Silk Road port cities.

Fourth, environmental sustainability integration is essential despite current framework limitations. The observed associations between rapid tourism growth and environmental pressures in cities like Colombo and Malacca underscore the necessity of incorporating ecological considerations into coordination assessment. Future policy frameworks should mandate environmental impact assessments, establish carrying capacity thresholds, and incentivize green tourism practices to ensure long-term sustainability.

### Methodological contributions and advantages

The multi-source remote sensing data integration approach provides unprecedented technical advantages for urban cultural tourism industry research, including objective measurement capabilities, continuous temporal coverage (annual from 2013, monthly for high-resolution data), and large-scale spatial monitoring that overcome traditional data limitations and subjective assessment biases^50^. The integration of Landsat, Sentinel-2, VIIRS nighttime light imagery, and synthetic aperture radar data enables comprehensive quantification of infrastructure development, tourism activity intensity, and economic performance indicators that support robust coupling coordination analysis.

The framework advances coupling coordination measurement methodologies through: (1) integration of remote sensing-derived indicators with socio-economic metrics, (2) development of standardized measurement protocols enabling cross-regional comparison, (3) implementation of spatial-temporal analytical techniques capturing dynamic evolution patterns, and (4) establishment of evidence-based policy recommendation frameworks. These methodological innovations contribute to broader academic discourse on sustainable urban development and cultural tourism integration.

### Conclusions

This research establishes a comprehensive analytical framework for evaluating spatial-temporal evolution of cultural tourism industry coupling coordination in Maritime Silk Road port cities using multi-source remote sensing data integration. The empirical analysis of 15 strategically important port cities during 2010–2020 reveals three distinct development phases (initial coordination establishment, rapid improvement, and stabilization optimization), significant spatial heterogeneity with regional clustering effects, and temporal convergence trends indicating narrowing developmental disparities.

The study demonstrates that economic foundation, transportation accessibility, and policy environment are strongly associated with coupling coordination outcomes, though definitive causal relationships require further investigation using quasi-experimental designs. Singapore, Hong Kong, and Shanghai exemplify mature integration models maintaining coordination degrees exceeding 0.85, while emerging economies like Gwadar, Djibouti, and Mombasa demonstrate rapid catch-up growth patterns with annual improvement rates above 7%, suggesting effectiveness of knowledge diffusion mechanisms across the Maritime Silk Road network.

The research contributes significant theoretical advances in coupling coordination measurement methodologies and spatial-temporal evolution analysis techniques, while providing evidence-based policy recommendations for sustainable cultural tourism industry development. The multi-source remote sensing approach offers scalable monitoring solutions adaptable across diverse geographical and cultural contexts, supporting informed decision-making for regional development planning, investment prioritization, and international cooperation strategies.

However, several important limitations constrain the current analysis and interpretation of findings. Data availability constraints before 2012, particularly the reliance on DMSP-OLS to VIIRS cross-calibration for 2010–2011 estimates, introduce ± 8–12% uncertainty in early-phase tourism intensity values, weakening conclusions about the initial coordination establishment phase. The absence of completed field validation represents a significant methodological gap, especially for informal tourism economies in data-scarce regions like Gwadar and Djibouti where remote sensing proxies may have ± 15–25% margin of error. The equal weighting assumption (α = β = 0.5) provides a standardized baseline for comparison but may not reflect context-specific priorities in all cities; while sensitivity analysis confirms ranking stability (Spearman ρ > 0.91), absolute coordination values vary by ± 0.08–0.15 units across different weight scenarios.

Environmental indicator integration remains limited in the current coupling coordination framework. While environmental degradation patterns are discussed qualitatively, ecological footprint and carbon emission metrics are not incorporated into the core coupling model due to data availability constraints across all 15 cities during 2010–2020. This omission is significant given observed correlations between tourism growth and NDVI decline in cities like Colombo and Malacca, though establishing causality requires more sophisticated analytical designs. The study’s cross-sectional and limited panel data structure constrains causal inference; associations between factors and coordination outcomes should not be interpreted as definitive causal relationships without quasi-experimental evidence.

Framework transferability to non-port city contexts remains uncertain. The coupling coordination model is specifically designed for port cities with dual maritime heritage and trade functions, incorporating port connectivity indices and coastal tourism infrastructure metrics that may not apply to inland tourism cities, non-BRI cities, or landlocked heritage sites. Preliminary transferability assessment shows that applying the framework to inland Chinese tourism cities (Lijiang, Zhangjiajie) without modification yields low predictive accuracy (r²=0.42 vs. 0.78 for port cities), confirming substantial methodological adaptation requirements for diverse urban typologies. Adaptation for non-port contexts necessitates replacing port connectivity with railway/highway accessibility metrics, substituting maritime heritage density with terrestrial cultural heritage indicators, and adjusting weight coefficients to reflect non-maritime tourism structures.

Future research should address these limitations through multiple pathways. First, field validation campaigns planned for 2024–2025 should verify remote sensing-derived indicators and incorporate results into revised frameworks with validated uncertainty bounds. Second, environmental metrics should be integrated into the core coupling coordination model to create a triple-bottom-line framework: ecological footprint per tourist using global NPP data, carbon emissions from tourism transport using shipping/aviation databases, coastal water quality monitoring using Sentinel-2/3 ocean color sensors, and green space connectivity indices using high-resolution land cover classification. This would align the framework with SDG targets for sustainable tourism development.

Third, advanced machine learning methodologies offer substantial potential for framework enhancement. Deep learning-based super-resolution fusion techniques such as Enhanced Deep Super-Resolution (EDSR) networks or Generative Adversarial Networks could address multi-sensor resolution discrepancies between Sentinel-2 (10 m) and VIIRS (500 m), enabling sub-100 m economic activity mapping. ResNet-50 or EfficientNet architectures for automated cultural facility identification from high-resolution imagery (e.g., Maxar WorldView-3 at 0.3 m resolution) could improve heritage density estimation accuracy by 35–50% compared to traditional object-based image analysis. Vision Transformer models show promise for capturing long-range spatial dependencies in urban tourism infrastructure patterns, while Spatio-temporal Graph Convolutional Networks could model inter-city coupling coordination dynamics by representing port cities as nodes and trade/tourism flows as edges, enabling prediction of coordination spillover effects across Maritime Silk Road networks.

Fourth, establishing definitive causal relationships requires employing panel data models with city fixed effects, difference-in-differences designs comparing BRI cities with matched non-BRI control cities, or synthetic control methods to isolate policy treatment effects from confounding trends. These quasi-experimental approaches would strengthen evidence for policy effectiveness and mechanism validation. Finally, developing an open-access web-based GIS platform disseminating coupling coordination degree metrics would enhance research impact and policy utility, allowing stakeholders to visualize spatial-temporal evolution patterns and conduct scenario analyses for evidence-based decision-making.

The findings provide robust foundations for targeted policy interventions promoting sustainable cultural tourism industry development across diverse geographical and economic contexts along the Maritime Silk Road, while acknowledging the need for continued methodological refinement and empirical validation to enhance framework reliability and applicability.

## Supplementary Information

Below is the link to the electronic supplementary material.

Supplementary Material 1

## Data Availability

All data generated and analyzed during the current study are available from the corresponding author upon reasonable request.
