# Supplementary material for: Spatial-temporal evolution analysis of cultural tourism industry coupling coordination in maritime silk road port cities: a multi-source remote sensing approach
Source: Sci Rep. 2025 Nov 27;15:42343. doi: 10.1038/s41598-025-26393-3 (PMC12660954; doi:10.1038/s41598-025-26393-3)
Supplement: Supplementary file 1 — Supplementary Material 1 [file 41598_2025_26393_MOESM1_ESM.docx]

# Supplementary Material

**Manuscript Title:** Spatial-Temporal Evolution Analysis of Cultural Tourism Industry Coupling Coordination in Maritime Silk Road Port Cities: A Multi-Source Remote Sensing Approach

**Corresponding Author:** Qianqian Xie (15797798865@163.com)

**Authors:** Dekun Liu, Qianqian Xie, Qiang Li, Yiyuan Wu

This supplementary material contains raw annual data and statistical analyses supporting the main manuscript findings.

## Table S1. Annual Coupling Coordination Degrees for Maritime Silk Road Port Cities (2010-2020)

| City Name | 2010 | 2011 | 2012 | 2013 | 2014 | 2015 | 2016 | 2017 | 2018 | 2019 | 2020 | Mean | Std Dev |
| --- | --- | --- | --- | --- | --- | --- | --- | --- | --- | --- | --- | --- | --- |
| Singapore | 0.823 | 0.834 | 0.845 | 0.851 | 0.862 | 0.874 | 0.883 | 0.891 | 0.896 | 0.902 | 0.907 | 0.867 | 0.028 |
| Hong Kong | 0.834 | 0.841 | 0.847 | 0.852 | 0.859 | 0.867 | 0.875 | 0.882 | 0.887 | 0.893 | 0.898 | 0.867 | 0.022 |
| Shanghai | 0.785 | 0.798 | 0.812 | 0.823 | 0.841 | 0.856 | 0.869 | 0.878 | 0.884 | 0.889 | 0.893 | 0.848 | 0.037 |
| Dubai | 0.698 | 0.721 | 0.743 | 0.762 | 0.789 | 0.813 | 0.834 | 0.851 | 0.863 | 0.872 | 0.878 | 0.802 | 0.060 |
| Venice | 0.756 | 0.762 | 0.768 | 0.773 | 0.781 | 0.789 | 0.796 | 0.802 | 0.806 | 0.809 | 0.811 | 0.787 | 0.019 |
| Mumbai | 0.612 | 0.634 | 0.656 | 0.675 | 0.701 | 0.726 | 0.748 | 0.767 | 0.782 | 0.794 | 0.803 | 0.718 | 0.063 |
| Piraeus | 0.598 | 0.615 | 0.631 | 0.645 | 0.667 | 0.687 | 0.705 | 0.721 | 0.734 | 0.744 | 0.752 | 0.681 | 0.052 |
| Alexandria | 0.567 | 0.584 | 0.600 | 0.614 | 0.635 | 0.654 | 0.671 | 0.686 | 0.698 | 0.708 | 0.716 | 0.648 | 0.050 |
| Thessaloniki | 0.534 | 0.548 | 0.561 | 0.573 | 0.591 | 0.607 | 0.622 | 0.635 | 0.646 | 0.655 | 0.662 | 0.603 | 0.042 |
| Malacca | 0.489 | 0.512 | 0.535 | 0.556 | 0.584 | 0.610 | 0.634 | 0.655 | 0.673 | 0.688 | 0.701 | 0.603 | 0.069 |
| Colombo | 0.456 | 0.478 | 0.499 | 0.519 | 0.547 | 0.574 | 0.598 | 0.620 | 0.639 | 0.655 | 0.669 | 0.568 | 0.069 |
| Mombasa | 0.398 | 0.423 | 0.447 | 0.470 | 0.502 | 0.533 | 0.562 | 0.588 | 0.612 | 0.633 | 0.652 | 0.529 | 0.080 |
| Dar es Salaam | 0.387 | 0.410 | 0.432 | 0.453 | 0.483 | 0.511 | 0.538 | 0.562 | 0.584 | 0.604 | 0.622 | 0.508 | 0.074 |
| Djibouti | 0.298 | 0.324 | 0.349 | 0.373 | 0.407 | 0.440 | 0.472 | 0.501 | 0.528 | 0.552 | 0.574 | 0.456 | 0.089 |
| Gwadar | 0.187 | 0.203 | 0.219 | 0.235 | 0.261 | 0.289 | 0.318 | 0.348 | 0.378 | 0.408 | 0.437 | 0.298 | 0.082 |

## Statistical Summary by Development Phases

### Phase 1 (Initial Coordination Establishment: 2010-2013)

- Mean coordination degree: 0.561
- Standard deviation: 0.208
- Coefficient of variation: 0.371
- Annual growth rate: 4.8%

### Phase 2 (Rapid Improvement: 2014-2017)

- Mean coordination degree: 0.661
- Standard deviation: 0.185
- Coefficient of variation: 0.280
- Annual growth rate: 7.2%

### Phase 3 (Stabilization Optimization: 2018-2020)

- Mean coordination degree: 0.712
- Standard deviation: 0.156
- Coefficient of variation: 0.219
- Annual growth rate: 3.9%

## Growth Rate Analysis by City Categories

### High Development Cities (Coordination > 0.8)

- Singapore: 2.01% annual growth
- Hong Kong: 1.87% annual growth
- Shanghai: 3.26% annual growth

### Rapid Growth Cities (Annual growth > 7%)

- Gwadar: 8.84% annual growth
- Djibouti: 6.78% annual growth
- Mombasa: 5.12% annual growth
- Dar es Salaam: 4.87% annual growth

**Note:** This raw annual data confirms the three-stage division pattern described in the main analysis and demonstrates the convergence trends, with the coefficient of variation decreasing from 0.371 in Phase 1 to 0.219 in Phase 3. The data supports the spatial-temporal evolution patterns presented in Figure 4 of the main manuscript, where 3-year moving averages were applied to reduce short-term volatility while preserving underlying development trends.
